# Influence of Explanatory Variable Distributions on the Behavior of the Impurity Measures Used in Classification Tree Learning

**DOI:** 10.3390/e26121020

**Published:** 2024-11-26

**Authors:** Krzysztof Gajowniczek, Marcin Dudziński

**Affiliations:** Institute of Information Technology, Warsaw University of Life Sciences-SGGW, 02-787 Warszawa, Poland; marcin_dudzinski@sggw.edu.pl

**Keywords:** decision trees, generalized entropy, imbalanced data, interactive learning, machine learning

## Abstract

The primary objective of our study is to analyze how the nature of explanatory variables influences the values and behavior of impurity measures, including the Shannon, Rényi, Tsallis, Sharma–Mittal, Sharma–Taneja, and Kapur entropies. Our analysis aims to use these measures in the interactive learning of decision trees, particularly in the tie-breaking situations where an expert needs to make a decision. We simulate the values of explanatory variables from various probability distributions in order to consider a wide range of variability and properties. These probability distributions include the normal, Cauchy, uniform, exponential, and two beta distributions. This research assumes that the values of the binary responses are generated from the logistic regression model. All of the six mentioned probability distributions of the explanatory variables are presented in the same graphical format. The first two graphs depict histograms of the explanatory variables values and their corresponding probabilities generated by a particular model. The remaining graphs present distinct impurity measures with different parameters. In order to examine and discuss the behavior of the obtained results, we conduct a sensitivity analysis of the algorithms with regard to the entropy parameter values. We also demonstrate how certain explanatory variables affect the process of interactive tree learning.

## 1. Introduction

### 1.1. Preliminary Information on Decision Trees

Decision Trees (DTs) are one of the most popular and powerful classification algorithms used in machine learning, data mining, and statistical analysis. They enable machine learning experts and data scientists to solve complex problems from various fields of interest. At the core of the DT algorithms lies the concept based on the application of disorder and uncertainty (impurity) measures, which provide guidance in the decision-making processes.

Initial DT studies were conducted in the mid-1930s by Charles J. Clopper and Egon S. Pearson [1], who introduced the concept of binary decision processes. However, the modern implementation of DTs in the area of machine learning systems began nearly five decades later in 1984, when Breiman et al. (see [2]) developed a DT induction algorithm (inducer) called Classification and Regression Tree (CART) in which concepts based on application of the Gini diversity index and binary splitting—also currently widely used in the DT construction—were proposed. In turn, Quinlan (see [3]) developed another DT induction algorithm known as Iterative Dichotomiser 3 (ID3). Later, Quinlan (see [4]) improved the ID3 algorithm by introducing the C4.5 approach. As an enhancement of C4.5, the C5.0 inducer was designed by Quinlan (see [5]). These developments, enhancements, and integration of the DT algorithms into ensemble methods, such as random forests and boosting algorithms, have recently strengthened their place as crucial algorithms in the machine learning field of study.

DTs are named after their tree-like structure. A DT is a classifier that is expressed as a recursive partition of the instance space, where by an instance, we mean a single observation $x^{T}$ of the input data. Consequently, the instance space is just the domain (input space, range) for $x^{T}$ (in other words, it is the space of all possible instances for some learning task). In attribute-value learning, the instance space is often depicted as a geometric space, where each dimension corresponds to a single input attribute (feature).

The DT structure consists of nodes that form a rooted tree. A node that has no incoming edge is called a root. The other nodes have exactly one incoming edge—among them, we have a group of nodes with outgoing edges, called internal or test nodes, and a group of nodes with no outgoing edges known as leaves or, alternatively, as terminal or decision nodes. Each internal node splits the instance space into two or more subspaces according to a certain function of the input attribute values. In the simplest and most frequent case, each test considers a single attribute, and the instance space is partitioned according to this attribute’s value. In the case of numeric (continuous) attributes, the condition refers to a range of the corresponding variable.

The objective that lies at the heart of the DT algorithms is to create a model that predicts the value (or class) of a target variable *Y* based on instances of several input variables. These DT-based algorithms have several advantages. Firstly, they can be easily visualized in order to gather an intuitive understanding of what the algorithms create, since they are usually represented as a flowchart-like structure in which every internal node is a logical test (called a split) and every leaf is a prediction. During the inference process, each observation from the instance space starts at the root and ends in one of the leaves, following a path that is completely clear and highly interpretable to the user. Moreover, the DT algorithms are flexible, in the sense that they can capture non-linear relationships between the input data features and outputs. Apart from their inherent clarity, transparency, interpretability, and flexibility, DT-based methods have several other advantages. Namely, they provide a non-parametric model where no assumptions on the data are required, since DT-based algorithms can directly handle both categorical and numerical data and eliminate the need for data preprocessing (which is often obligatory in traditional statistical methods that frequently struggle with categorical variables, requiring them to be converted into numerical values). In particular, DT methods do not impose any assumptions regarding distribution, independence, or homogeneity of the underlying data, which is especially vital in applications where very little is known about the data and features used for predictions. Another significant aspect of DT-based algorithms is the fact that they display relatively low computational cost, which guaranties that tree-based decision rules for large datasets may be generated relatively quickly. This additionally allows the algorithm to consider data with missing values. Taken together, these aspects make DT algorithms stand out from other methods due to their versatility, efficiency, and utility in a variety of applications.

DTs are divided into two main types: (a) classification trees—where the predicted outcome of a target variable is a discrete class, which means that the algorithm classifies data points into different classes with remarkable accuracy, (b) regression trees—where the predicted outcome of a target variable is a real number, which means that the algorithm predicts the value of a continuous target variable by recursively splitting the data and fitting regression models to the established subsets.

### 1.2. Preliminary Overview of Decision Tree Induction Algorithms

As previously mentioned, each DT divides the whole instance space (input data space) into several subsets (subgroups) containing instances with (almost) the same predicted classes or values of a target valuable Y. In general, a DT consists of parent nodes and child nodes, where a child node is a node with an incoming edge (branch) from an outgoing edge (branch) of a parent node. The data aggregated in the parent nodes are partitioned into the smaller datasets that are aggregated in the child nodes (subsets). These datasets are obtained using the best possible input attributes (variables, features) that are selected by the specific splitting criteria (rules). These are mainly the so-called impurity-based criteria, which means that they involve employing appropriate indices for measuring the uncertainty or disorder within a dataset, such as information gain, gain ratio, the Gini index, and the twoing rule, among others.

DT inducers are algorithms that automatically construct a DT from a given dataset. Their purpose is to construct an optimal DT by minimizing the suitable generalization error, although other target functions, e.g., the functions minimizing the number of nodes or an average depth of a tree, may be taken into account in this context. However, it has been shown that finding a minimal DT that is consistent with the provided training set is NP-hard (see [6]). This implies that using optimal DT algorithms is appropriate only in small problems. As a result, heuristic methods need to be applied in order to overcome this drawback. These methods can be divided into two groups—top-down and bottom-up. The first of the mentioned groups is preferred in contemporary research works. Top-down induction is a recursive method of DT generation that starts with the entire input dataset in the root node, where a locally optimal test for data splitting is searched, and branches corresponding to the test outcomes are created. Then, the data splitting procedures are repeated in the created nodes unless the stopping condition is fulfilled (the splitting process is stopped when there is only a single case in each of the terminal nodes or when all cases within each terminal node have the same distribution of predictor variables, making splitting impossible).

Numerous top-down DT inducers have been introduced in recent decades. Among them, DT induction algorithms including CART [2], ID3 [3], C4.5 [4], and C5.0 [5] are particularly worthwhile to mention, since they have had an enormous impact on the development and enhancement of DT-based algorithms. Most DT induction algorithms perform classification tasks in two conceptual phases: tree-growing and tree-pruning (e.g., C4.5 and CART). The other inducers perform only the tree-growing phase.

The tree-growing (building) phase has already been thoroughly described in the previous paragraphs. In the following subsection, the most popular splitting criteria for the tree-building, based on the application of impurity measures, are presented.

The tree-pruning phase can be divided into the pre-pruning and the post-pruning stages. Pre-pruning is used to limit the size of the tree and to stop the tree from fully growing and consequently to prevent the possibility of overfitting (see [7,8] for details). Due to the construction of smaller trees, pre-pruning provides a simple and computationally low-cost procedure in building the optimal DT. On the other hand, applying the pre-pruning parameters too aggressively may result in underfitting. In turn, contrary to the pre-pruning, the post-pruning technique, also known as backward pruning, enables the tree to grow to its full size at first, then prunes it back (see [9]). The most common methods applied for DT pruning include techniques such as cost complexity pruning, reduced error pruning, pessimistic pruning, minimum error pruning, and error-based pruning.

### 1.3. Primary Goals and Conceptions

Decision trees are typically constructed by specifying certain parameters, running the algorithm, and evaluating the resulting tree structure. After the learning parameters are adjusted, the algorithm is re-run. This process continues until the user is satisfied with the decision tree. In many cases, the experts who create decision trees have extensive knowledge on their fields but may not be well-versed in the algorithms underlying a decision tree’s building process. As a result, they may not fully understand the specific meanings of parameters and their impact on the resulting decision tree. Likewise, they may have limited knowledge about the inner workings of the employed algorithms, which often leads to the construction of a decision tree becoming a trial-and-error process that can be quite time-consuming. Furthermore, domain experts may not be able to use their knowledge of how to optimize a decision tree because the algorithm may act as a black box that they cannot control.

According to [10,11], it is important to use expert knowledge and employ visualization of the modeling process. This is done for several reasons, summarized as follows:

- Delivering appropriate data and knowledge visualization can make use of human pattern recognition capabilities to enhance the effectiveness of a decision tree’s construction process;
- Through active involvement of visualization techniques, experts can gain a deeper understanding of the resulting decision tree;
- By obtaining indirect results from the corresponding algorithm, experts can contribute to the development of domain knowledge (e.g., important variables), resulting in further exploration of the algorithm. Using of expert knowledge has been recognized as a promising approach leading to reductions in computational costs and the avoidance of overfitting;
- An interactive design process improves the performance of the model, makes the algorithm more understandable for users, and simultaneously increases satisfaction arising from solving the stated problem.

The industry has recognized the importance of incorporating expert knowledge into automated decision tree generation. It is believed that interactive learning can fill this gap. Recently, several new methods (see [12,13,14]) have emerged in order to encourage users to apply more intensive work on data exploration and visualization methods. This approach is known as visual data exploration. We present the *ImbTreeEntropy* package [15,16,17] (available at https://github.com/KrzyGajow/ImbTreeEntropy, accessed on 14 October 2024) in this context. This package combines automatic algorithms, interactive algorithms, and visualization methods.

The primary objective of our study is to analyze how the nature of explanatory variables influences the values and behavior of impurity measures, including the Shannon, Rény, Tsallis, Sharma–Mittal, Sharma–Taneja, and Kapur entropies. Our analysis uses the mentioned measures in interactive learning of decision trees, particularly in tie-breaking situations where an expert needs to make a decision. We simulated the explanatory variables from various probability distributions in order to encompass a wide range of variability and properties. These distributions include the normal, uniform, Cauchy, exponential, Beta and Beta2 probability distributions.

The main contributions of this article may be summarized as follows:

- Our study shows the relationships between the impurity measures and the nature of explanatory variables used in the considered model;
- Our analysis indicates which entropy parameter values are not feasible for DT training;
- We employ a large collection of generalized entropy functions, including the Rényi, Tsallis, Sharma–Mittal, Sharma–Taneja, and Kapur entropies, as the impurity measures of the tree nodes in our *ImbTreeEntropy* algorithm;
- We implement an interactive learning process in order to enable experts to make decisions regarding the selection of an optimal split in ambiguous situations;
- We implement an interactive learning process that allows for the construction of a completely new tree from scratch by incorporating the specific knowledge provided by an expert;
- We show which of the applied impurity measures are preferred in interactive DT learning depending on the nature of the explanatory variables.

To summarize our paper’s primary objectives, we aim to analyze how the nature of explanatory variables influences the values and behavior of distinct impurity measures that are used in the interactive learning of trees in tie-breaking situations where an expert has to make the ultimate decision. For this reason, it is not possible to compare the obtained results, or rather their quality, with other methods. However, this is not a drawback, since such a comparison is not the main goal of our design.

The remainder of our paper is organized as follows: Section 2 presents an overview concerning the subject of impurity measures; Section 3 gives a theoretical background on the entropy measures used in our research; Section 4 presents a comparative study of the existing DT induction algorithms; Section 5 presents the probability distributions applied in our further simulations; Section 6 outlines the conducted experiments, and it discusses and comments on the obtained results; Section 7 concludes our investigations.

## 2. Literature Review and Theoretical Background

### 2.1. General Definition and Properties of Impurity Measures

The splitting criteria, also known as the splitting rules, describe the methods that enable us to determine where a tree should be split in its nodes and how to divide the dataset into appropriate subsets of observations. The selection of the splitting criterion is vital, as it directly influences both the tree’s structure and its performance. In the majority of cases, the splitting functions are univariate, which means that an internal node is split according to the value of a single attribute (consequently, the best attribute upon which to split is searched). Various univariate criteria exist, and different DT algorithms employ different splitting criteria. An overview of the most well-known splitting rules is given in later sections of this article. As the essence of these criteria involves the use of impurity measures, we will start with an introduction of the general definition and meaning of impurity measures.

Suppose now that we have a discrete (categorical) random variable *Y* with *k* possible values (labels, classes): $c_{1},c_{2},\ldots ,c_{k}$, and that it has a distribution $\left\{\left(c_{1},p_{1}\right),\left(c_{2},p_{2}\right)\ldots ,\left(c_{k},p_{k}\right)\right\}$, where $p_{l}=P\left(Y=c_{l}\right),\;l=1,2,\ldots ,k$. In addition, $P=P_{Y}$ stands for the probability vector ${\left[p_{1},p_{2},\ldots ,p_{k}\right]}^{T}$. Then, an impurity measure of *Y* is a function $\Phi :{\left[0,1\right]}^{k}\to R$ satisfying the following properties (see [18]):

- Φ(P) takes non-negative values,
- Φ(P) attains a minimum value of 0 if $p_{l_{0}}=1$ for some $l_{0}$, and ${p_{l}}^{\prime }s$ are zero for all $l\neq l_{0}$,
- Φ(P) attains its maximum value if $p_{1}=p_{2}=\ldots =p_{k}=\frac{1}{k}$,
- Φ(P) is symmetric with respect to the components of the probability vector *P*,
- Φ(P) is everywhere differentiable in its range.

Let *S* denote the training set—i.e., the set of input values from the space $X^{p}=\left\{{\left[X_{1},\ldots ,X_{p}\right]}^{T}\right\}$ of random vectors, and *Y* be a target (response) random variable in our model. As above, we assume that *Y* is discrete (categorical) with classes $\left\{c_{1},c_{2},\ldots ,c_{\left|domain(Y)\right|}\right\}$. Then, the probability vector of *Y* is given as follows:

(1) $$P_{Y}\left(S\right)={\left[\frac{|\delta_{\left\{Y=c_{1}\right\}}S|}{\left|S\right|},\frac{|\delta_{\left\{Y=c_{2}\right\}}S|}{\left|S\right|},\ldots ,\frac{|\delta_{\left\{Y=c_{\left|domain(Y)\right|}\right\}}S|}{\left|S\right|}\right]}^{T},$$

where |·| stands for the cardinality of a given set, and (for l=1,2,…,|domain(Y)|), $\left\{\delta_{\left\{Y=c_{l}\right\}}S\right\}$ denotes the subset of those instances (observations) from the training set *S* which belong to the *l*-th class of *Y*.

We are now in a position to define the goodness-of-split for the attribute $X_{i}$, i=1,2,…,p, as the expected reduction in impurity of a target variable *Y* after partitioning *S* according to the values of $X_{i}$. It is formulated as follows (see [18]):

(2) $$\Delta \Phi \left(X_{i},S\right)=\Phi \left(P_{Y}\left(S\right)\right)-\sum_{v_{i,j}\in domain(X_{i})}\;\frac{|\delta_{\left\{X_{i}=v_{i,j}\right\}}S|}{\left|S\right|}\;\Phi (P_{Y}\left(\delta_{\left\{X_{i}=v_{i,j}\right\}}S\right)),$$

where (for $j=1,2,\ldots ,|domain(X_{i})|$) $\left\{\delta_{\left\{X_{i}=v_{i,j}\right\}}S\right\}$ stands for the subset of those instances (observations) from the training set *S*.

### 2.2. Main Impurity Measures-Based Rules (Criteria)

Except for some minor changes in notation, most of the formulas below have been incorporated based on [18].

#### 2.2.1. Information Gain as a General Impurity-Based Measure

The information gain is a criterion applied in the ID3 and C4.5 induction algorithms. It is the impurity-based criterion where the notion of entropy in information theory is used as the impurity measure Φ. Thus, it is defined as follows (see [18]):

(3) $$InformationGain\left(X_{i},S\right)=Entropy\left(Y,S\right)-\sum_{v_{i,j}\in domain(X_{i})}\;\frac{|\delta_{\left\{X_{i}=v_{i,j}\right\}}S|}{\left|S\right|}\;Entropy\left(Y,\delta_{\left\{X_{i}=v_{i,j}\right\}}S\right),$$

where:

(4) $$Entropy\left(Y,S\right)=-\sum_{c_{l}\in domain(Y)}\;\frac{|\delta_{\left\{Y=c_{l}\right\}}S|}{\left|S\right|}\;\log_{2}\;\frac{|\delta_{\left\{Y=c_{l}\right\}}S|}{\left|S\right|}.$$

#### 2.2.2. Gini Gain

The Gini gain is a splitting criterion applied in the CART induction algorithm. It is the impurity-based criterion where the Gini index (also known as the Gini impurity) is utilized as the impurity measure Φ. Hence, the Gini gain of selecting a feature $X_{i}$ is determined by the following formula (see [18]):

(5) $$GiniGain\left(X_{i},S\right)=Gini\left(Y,S\right)-\sum_{v_{i,j}\in domain(X_{i})}\;\frac{|\delta_{\left\{X_{i}=v_{i,j}\right\}}S|}{\left|S\right|}\;Gini\left(Y,\delta_{\left\{X_{i}=v_{i,j}\right\}}S\right),$$

where:

(6) $$Gini\left(Y,S\right)=1-\sum_{c_{l}\in domain(Y)}\;{\left(\frac{|\delta_{\left\{Y=c_{l}\right\}}S|}{\left|S\right|}\right)}^{2}.$$

#### 2.2.3. DKM Rule for Binary Classes

The DKM splitting criterion, named after its authors, Dietterich, Kearns, and Mansour (see [19,20]), has been designed for the binary target variable *Y* (i.e., for the variable where $domain\left(Y\right)=\left\{c_{1},c_{2}\right\}$). This criterion has several advantages. Namely, the authors have shown that for a given level of the prediction accuracy, the expected size of a DKM-based tree is smaller than for the trees constructed by using the C4.5 or Gini-based algorithms. The corresponding impurity-based function is given as follows (see [18]):

(7) $$DKM\left(Y,S\right)=2\cdot \sqrt{\frac{|\delta_{\left\{Y=c_{1}\right\}}S|}{\left|S\right|}\cdot \frac{|\delta_{\left\{Y=c_{2}\right\}}S|}{\left|S\right|}}.$$

### 2.3. Selected Normalized Impurity Measures-Based Rules (Criteria)

The previously presented impurity-based criteria are biased, in the sense that they prefer attributes (features) with larger numbers of distinct values. Although adding such attributes into a DT may result in an increase in the decision tree’s information gain, it may simultaneously cause a decrease of the tree’s generalized accuracy. In order to reduce this problem, normalization of the impurity-based measures is conducted. This normalization is achieved by dividing the information gain by the suitable split information measure.

#### 2.3.1. Gain Ratio

The gain ratio is a normalized splitting criterion that is primarily used in the C4.5 decision tree induction algorithm in order to reduce bias of the information gain towards multi-valued features. It ultimately leads to a more balanced and effective DT. The gain ratio is calculated as follows (see [18]):

(8) $$GainRatio\left(X_{i},S\right)=\frac{InformationGain(X_{i},S)}{Entropy(X_{i},S)}.$$

#### 2.3.2. Distance Measure

The distance measure is another criterion that normalizes an appropriate impurity measure, but this normalizing is carried out in a different manner. Generally, it is computed according to the following formula (see [18]):   

(9) $$DistanceMeasure\left(X_{i},S\right)=-\frac{\Delta \Phi (X_{i},S)}{\sum_{v_{i,j}\in domain(X_{i})}\sum_{c_{l}\in domain(Y)}\frac{|\delta_{\left\{X_{i}=v_{i,j},Y=c_{l}\right\}}S|}{\left|S\right|}\log_{2}\;\frac{|\delta_{\left\{X_{i}=v_{i,j},Y=c_{l}\right\}}S|}{\left|S\right|}}.$$

### 2.4. Binary Impurity Measures-Based Rules (Criteria)

Binary rules are applied for building binary DTs, i.e., for the trees designed based on the division of the input attribute domain into two sub-domains. Binary decision trees are often used, mainly because many attributes are naturally binary, binary trees are easy to interpret, and various mathematical properties can be implemented in a binary architecture.

#### 2.4.1. Twoing Rule

This binary criterion is expressed as follows (see [18]):

(10) $$\begin{array}{c} Twoing\left(X_{i},domain_{1}\left(X_{i}\right),domain_{2}\left(X_{i}\right),S\right)=0.25\cdot \frac{|\delta_{\left\{X_{i}\in domain_{1}\left(X_{i}\right)\right\}}S|}{\left|S\right|}\cdot \frac{|\delta_{\left\{X_{i}\in domain_{2}\left(X_{i}\right)\right\}}S|}{\left|S\right|}\\ \cdot {\left(\sum_{c_{l}\in domain(Y)}\;\left|\frac{|\delta_{\left\{X_{i}\in domain_{1}\left(X_{i}\right),Y=c_{l}\right\}}S|}{|\delta_{\left\{X_{i}\in domain_{1}\left(X_{i}\right)\right\}}S|}-\frac{|\delta_{\left\{X_{i}\in domain_{2}\left(X_{i}\right),Y=c_{l}\right\}}S|}{|\delta_{\left\{X_{i}\in domain_{2}\left(X_{i}\right)\right\}}S|}\right|\right)}^{2}. \end{array}$$

#### 2.4.2. Orthogonal Rule

This binary criterion has been proposed in [21]. It is defined as follows (see [18]):

(11) $$Orthogonal\left(X_{i},domain_{1}\left(X_{i}\right),domain_{2}\left(X_{i}\right),S\right)=1-\cos\left(∠\left(P_{Y,1},P_{Y,2}\right)\right),$$

where $∠(P_{Y,1},P_{Y,2})$ denotes an angle between $P_{Y,1}$ and $P_{Y,2}$, representing the distribution of a target variable *Y* in the subsets (partitions) $\left\{\delta_{X_{i}\in domain_{1}\left(X_{i}\right)}S\right\}$ and $\left\{\delta_{X_{i}\in domain_{2}\left(X_{i}\right)}S\right\}$ of the training set *S*, respectively.

#### 2.4.3. Kolmogorov–Smirnov Rule

This binary criterion uses the Kolmogorov–Smirnov distance and has been introduced in [22,23]. The Kolmogorov–Smirnov criterion requires that the domain of a target attribute *Y* is binary (i.e., $domain\left(Y\right)=\left\{c_{1},c_{2}\right\}$). It is determined as follows (see [18]):

(12) $$\begin{array}{c} KolmogorovSmirnov(X_{i},domain_{1}\left(X_{i}\right),domain_{2}\left(X_{i}\right),S)=\\ \left|\frac{|\delta_{\left\{X_{i}\in domain_{1}\left(X_{i}\right),Y=c_{1}\right\}}S|}{|\delta_{\left\{Y=c_{1}\right\}}S|}-\frac{|\delta_{\left\{X_{i}\in domain_{2}\left(X_{i}\right),Y=c_{2}\right\}}S|}{|\delta_{\left\{Y=c_{2}\right\}}S|}\right|. \end{array}$$

### 2.5. Interactive Learning

By default, the algorithm presented in [16] automatically constructs a tree. However, there are three different types of interactive learning, allowing for the construction of an entire tree from scratch and simultaneously allowing an expert to make decisions directly during each division. The other two types of interactive learning only enable experts to make decisions in ambiguous situations.

The question arises of how ambiguity should be defined. In order to explain it, we consider examples with two meanings of ambiguity. In the first case, we assume that we have a multiclass classification problem with four classes, and we wish to divide a particular node. Suppose that in the left child node, the estimated probabilities of two classes assigned by a particular node are similar, e.g., they are equal to 0.44 and 0.46, and that very small probabilities, e.g., 0.06 and 0.04, are assigned to the other classes. In addition, in the second node, the corresponding probabilities are 0.48, 0.47, 0.02, and 0.03. An algorithm chooses the second class (left child) or the first class (right child) as the label. The point is that when the data are divided, one observation can significantly influence the corresponding probabilities and even the final classification of the class. For an expert, it might be better to choose a different division (a different cut-off point or a different attribute), even if it results in a lower information gain or gain ratio, because it provides a clearer difference in probabilities. Therefore, if an algorithm produces a division that significantly favors one class (a simple decision), we keep it as it is. Only when there are divisions with questionable probabilities, an expert will have to interfere in making the ultimate decision.

The second meaning of ambiguity is related to the frequencies of observations of the class in a node. Assume that we have four classes and that their frequencies in a dataset consisting of 100 observations are 10 (10%), 15 (15%), 35 (35%), and 40 (40%). Based on this, we aim to focus our attention on the second class, and we wish to build a model that can classify this class accurately. We may encounter situations where the best division obtained trough maximization of the information gain or the gain ratio splits the observations of the considered class into both child nodes. In interactive learning, an expert can specify which classes are important and how many observations (in terms of frequencies) should be present in a node to make a decision. If these thresholds are not determined, then an algorithm will make decisions based on the best division. For example, if an expert sets thresholds of 50%, 0%, 100%, and 100%, it means that a decision will be made only when there are more than 50 instances in the first class, any instances in the second class, 35 instances in the third class, and 40 instances in the fourth class. This implies that the third and fourth classes have no impact on the decision-making process, while any observation in the second class will prompt a decision.

### 2.6. Other Entropies

It is also vital to mention the notion of fractional entropies in our literature review. The concept of fractional entropies is based on the application of fractional calculus. For a comprehensive overview regarding fractional entropies and their implementation, we refer to the papers by Lopes and Machado [24], Machado et al. [25,26], Akimoto and Suzuki [27], Ubriaco [28], Radhakrishnan et al. [29], and Karci [30]. In turn, among the recent works where fractional entropies are used in DT learning, the publications by Suthaharan [31] and De la Cruz-García et al. [32] are especially worth mentioning. We also contribute information regarding the application of Dempster–Shafer evidence theory to DT learning in our literature review. Dempster–Shafer theory owes its name to the papers by Dempster [33] and Shafer [34]. This theory attracted attention in the early 1980s when AI researchers were trying to adapt probability theory to expert and recommendation systems. It is also known as the theory of belief functions and is a generalization of Bayesian theory from subjective probability. When it comes to the application of Dempster–Shafer theory in the DT learning, it should be emphasized that although DTs are efficient classification techniques in data mining, typical DT algorithms perform suboptimally when dealing with data showing uncertainties both at the construction and classification stages. Dempster–Shafer theory offers an alternative approach to traditional probabilistic theory for the mathematical representation of these uncertainties. Li et al. [35] have shown that DT techniques can be extended to uncertain environment by employing Dempster–Shafer evidence theory. For the latest developments in areas related to the use of this theory, see the work by Peñafiel et al. [36].

## 3. Applied Entropy Measures

The concept of entropy is a common expression used in various fields of interest, including thermodynamics, statistical physics, and information theory. With regard to the last of the mentioned areas, entropy of information of a given random variable is the amount of information contained in this random variable. By this, we mean the amount of information that may be gained by the variable’s outcomes in disordered systems, which is interpreted as an average level of unpredictability and uncertainty (i.e., as an average level of disorder) of the events connected with the corresponding variable. In the definition of information entropy, an event that is certain (i.e., has a probability of 1) has an entropy of 0, since it provides no new information, whereas if an outcome of the event is completely uncertain, then the corresponding entropy reaches its maximum. For example, tossing a fair coin with two possible outcomes (heads or tails), each with a probability of occurrence equal to 0.5, results in an entropy of 1 bit.

The idea of information entropy originates from the concept of entropy in physics, which aims to describe the disorder of physical systems. It was introduced by Shannon [37] (see also [38]), where the first measure of uncertainty of the random variable—commonly known as the Shannon entropy—was proposed. Before we define the Shannon entropy, we will introduce some additional notations.

### 3.1. Notation

Let us consider the supervised learning problem with a structured set of *n* labeled data points ${\left\{\left(x_{s}^{T},y_{s}\right)\right\}}_{s=1,\ldots ,n}$, where $x_{s}^{T}\in X^{p}$ denotes the vector of feature values for the *s*-th object from an available set of elements and $X^{p}=\left\{{\left[X_{1},\ldots ,X_{p}\right]}^{T}\right\}$ stands for a *p*-dimensional feature space. Simultaneously, $y_{i}$s are either real empirical values of the regression function or the labels (classes) of some categorical target (response) random variable *Y*. In the process of supervised learning, the observations $\left(x_{s}^{T},y_{s}\right)$ form the training dataset *S*. Based on this training set, the supervised learning methods aim to construct a model that finds a discriminant function which can successfully predict the label $y^{*}$ of a target valuable *Y* for the new input feature vector $x^{*T}$.

Since we aim to use the logistic model in further research, we assume that *Y* is a discrete random variable with the following classes: $c_{1},c_{2}\ldots ,c_{\left|domain(Y)\right|}$. In addition, we define the probabilities $p_{l}$ as follows:

(13) $$p_{l}=\frac{|\delta_{\left\{Y=c_{l}\right\}}S|}{\left|S\right|},\;\;l=1,2,\ldots ,\left|domain(Y)\right|,$$

where, for recollection, $\left\{\delta_{\left\{Y=c_{l}\right\}}S\right\}$ denotes the subset of those instances (observations) from the training set *S* which belong to the *l*-th class of a target (response) variable *Y*.

We are now in a position to define the entropy measures used in our simulation study.

### 3.2. Shannon Entropy

The Shannon entropy (type = “Shannon” in *ImbTreeEntropy*) is given by the following formula (see also [39]):

(14) $$H^{Sh}\left(Y,S\right)=-\sum_{l=1}^{\left|domain(Y)\right|}\;p_{l}\;\log_{2}\;p_{l}.$$

The Shannon entropy, named in honor of its founder, Claude Shannon, is a specific form of the information entropy. Its introduction in the mid-20th century paved the way for the rapid development of information theory, which not only played a crucial role in the understanding of information communication, transmission, storage, and processing but also had an invaluable influence on modern technology. This started with advancements in data transmission over the internet through the development of satellite technology and smartphones, as well as providing substantial support in creating the machine learning algorithms that have allowed for an expansion of cryptography techniques.

The Shannon entropy has a number of significant properties, namely (see, e.g., [40]):

- it takes non-negative values,
- it equals 0 if $p_{l_{0}}=1$ for some $l_{0}$, and ${p_{l}}^{\prime }s$ are zero for all $l\neq l_{0}$,
- it attains a maximum value if $p_{1}=\ldots =p_{\left|domain(Y)\right|}$,
- it is a concave function.

The Shannon entropy assumes some implicit trade-off between contributions from the tails and the main mass of the underlying variable distribution. It is of vital importance to control this trade-off explicitly. That has become possible by introducing the entropy measures depending on the powers of the probabilities $\left(\left\{{p_{l}}^{q}\right\}\right)$. Rényi [41] proposed a generalization of Shannon entropy (14), which is called the Rényi entropy.

### 3.3. Rényi Entropy

The Rényi entropy (type = “Renyi” in *ImbTreeEntropy*) is defined as follows (see also [39]):

(15) $$H_{q}^{R}\left(Y,S\right)=\frac{1}{1-q}\log_{2}\left(\sum_{l=1}^{\left|domain(Y)\right|}\;{p_{l}}^{q}\right),$$

where q≥0 and q≠1.

The Rényi entropy has similar properties to the Shannon entropy, but it possesses an additional parameter *q*, which can be used to make it more or less sensitive to the shape of probability distributions. If q∈(0,1), then the Rényi entropy is a concave function, while if q∈(1,∞), then it may be a concave or a convex function. In addition, when q→1, then the Rényi entropy is close to the Shannon entropy. Furthermore, it is also worth mentioning that provided $q_{1}\in \left(0,1\right)$ and $q_{2}\in \left(1,\infty \right)$, the following relation between the Rényi and Shannon entropies holds: $H_{q_{1}}^{R}\left(Y,S\right)\geq H^{Sh}\left(Y,S\right)\geq H_{q_{2}}^{R}\left(Y,S\right)$.

Apart from the Rényi entropy, another generalization of order *q* of the Shannon entropy has been proposed in [42]. It is called the Tsallis entropy.

### 3.4. Tsallis Entropy

The Tsallis entropy (type = “Tsallis” in *ImbTreeEntropy*) is determined as follows (see also [39]):

(16) $$H_{q}^{T}\left(Y,S\right)=\frac{1}{1-q}\left(\sum_{l=1}^{\left|domain(Y)\right|}\;{p_{l}}^{q}-1\right)=\frac{1}{q-1}\left(1-\sum_{l=1}^{\left|domain(Y)\right|}\;{p_{l}}^{q}\right).$$

Except for the earlier listed properties of the Shannon entropy, the Tsallis entropy satisfies the following additional properties:

- if $q_{1}<q_{2}$, then $H_{q_{1}}^{T}\left(Y,S\right)>H_{q_{2}}^{R}\left(Y,S\right)$,
- if q→1, then $H_{q}^{T}\left(Y,S\right)\to \left(\ln2\right)\cdot H^{Sh}\left(Y,S\right)$,
- the following relations between the Tsallis and Rényi entropies hold:
  $$H_{q}^{T}\left(Y,S\right)=\frac{1}{1-q}\left(e^{\left(1-q\right)H_{q}^{R}\left(Y,S\right)}-1\right),$$
  $$H_{q}^{R}\left(Y,S\right)=\frac{1}{1-q}\ln\left(1+\left(1-q\right)H_{q}^{T}\left(Y,S\right)\right).$$

The Rényi and Tsallis entropies are not mutual generalizations of each other. In order to partially fill this gap, two-parametric entropy measures have been introduced. They are known as the Sharma–Mittal, Sharma–Taneja, and Kapur entropies.

### 3.5. Sharma-Mittal Entropy

The Sharma–Mittal entropy (type = “Sharma-Mittal” in *ImbTreeEntropy*) is given as follows:

(17) $$H_{q,r}^{Sh-M}\left(Y,S\right)=\frac{1}{1-r}\left({\left(\sum_{l=1}^{\left|domain(Y)\right|}\;p_{l}^{q}\right)}^{\frac{1-r}{1-q}}\right).$$

### 3.6. Sharma–Taneja Entropy

The Sharma—Taneja entropy (type = “Sharma-Taneja” in *ImbTreeEntropy*) is defined as follows:   

(18) $$H_{\alpha ,\beta }^{Sh-T}\left(Y,S\right)={\left(2^{1-\alpha }-2^{1-\beta }\right)}^{-1}\left(\sum_{l=1}^{\left|domain(Y)\right|}\;p_{l}^{\alpha }-\sum_{l=1}^{\left|domain(Y)\right|}\;p_{l}^{\beta }\right),$$

where α≠β, α>0, β>0.

### 3.7. Kapur Entropy

Kapur entropy (type = “Kapur” in *ImbTreeEntropy*) is determined as follows:

(19) $$H_{\alpha ,\beta }^{K}\left(Y,S\right)=\frac{1}{1-\alpha }\ln\left(\sum_{l=1}^{\left|domain(Y)\right|}\;\frac{p_{l}^{\alpha +\beta -1}}{p_{l}^{\beta }}\right),$$

where α≠1, α>0, β>0, α+β−1>0.

It is worthwhile to mention that:

- if β→1, then the Sharma-Taneja entropy is close to the Tsallis entropy,
- if β=1, then the Kapur entropy reduces to the Rényi entropy,
- if β=1 and α→1, then the Kapur entropy is close to the Shannon entropy.

We also consider the Gini coefficient and the misclassification error in our research. The Gini coefficient (or index) is the most commonly used measure of inequality. It is typically applied as a measure of income inequality, but it can be implemented to measure the inequality of any distribution, e.g., the distribution of wealth or even life expectancy ([43]).

### 3.8. Gini Coefficient

Incorporating the notation for ${\left\{p_{l}\right\}}_{l=1,\ldots ,|domain(Y)|}$ from (13) (as was the case in Formulas (14)–(19)), we determine the Gini coefficient using the following formula:

(20) $$Gini\left(Y,S\right)=2\cdot \left(1-\sum_{l=1}^{\left|domain(Y)\right|}\;{p_{l}}^{2}\right).$$

### 3.9. Missclassification Error

The Missclassification Error (MCE) is expressed as follows:

(21) $$MCE\left(Y,S\right)=2\cdot \left(1-\max_{1\leq l\leq \left|domain(Y)\right|}p_{l}\right).$$

## 4. Comparative Study of the DT Induction Algorithms

Table 1 below contains a brief summary of the most commonly used DT inducers. The first four of the listed algorithms have been mentioned earlier: [2] (CART), [3] (ID3), [4] (C4.5), and [5] (C5.0). In turn, the idea of the CHAID (Chi-Square Automatic Interaction Detection) algorithm comes from [44].

## 5. Probability Distributions Applied in the Research Study

In this section, we aim to present the probability distributions of random variables, which we will later employ in our scientific research. Our main objective is to examine the relationships between the impurity measures used in the machine learning algorithms and the probability distributions of the explanatory random variables from the applied statistical models. We will only take the univariate distributions into account.

In our experiments, we have used a wide range of distributions. This range should be understood not only in the sense of the selected number of distribution types but more importantly in the sense of a large range of possible values for the parameters of the considered distributions, as well as in the sense of the adopted versatility of other characteristics describing the properties of these distributions (e.g., whether they are left-skewed or right-skewed, if they are unimodal or multimodal, etc.). We claim that the six distributions, with a relatively large number of assumed distribution parameters and considered characteristics and with different probabilistic and statistical properties, guarantee a necessary diversity of distributions selected for our simulation study, providing well-balanced results.

### 5.1. Normal Distribution

The normal distribution, equivalently known as the Gaussian distribution, is the most frequently used probability distribution in the areas of probability theory and statistical methods. It is mainly due to the normal distribution’s unique mathematical properties which make it applicable to many practical problems from various fields of interest. The normal probability distribution is the distribution of a random variable of a continuous type. Consequently, it is defined by determining its probability density function. Thus, a (univariate) normal probability distribution is a two-parameter distribution with a density function of the following form:

(22) $$f\left(x;\mu ,\sigma \right)=\frac{1}{\sqrt{2\pi }\;\sigma }e^{-\frac{{\left(x-\mu \right)}^{2}}{2\sigma^{2}}},\;\;x\in R,\;\mu \in R,\;\sigma >0,$$

where μ and σ (called the mean and the standard deviation, respectively) stand for the distribution parameters.

In other words, we say that a (univariate) random variable *X* has a normal distribution with the parameters μ and σ (which we symbolically denote as X∼N(μ,σ)) if its probability density function is given by the formula in (22).

If μ=0 and σ=1, then we are dealing with the so-called standard normal distribution. In this case, the probability density function from (22) clearly reduces to the following:

(23) $$f\left(x\right)=\frac{1}{\sqrt{2\pi }}e^{-\frac{x^{2}}{2}}.$$

### 5.2. Cauchy Distribution

In our simulation study, we will also consider the case when explanatory (input) variables have a Cauchy distribution. This is a distribution of a continuous type with a density function determined by the following:

(24) $$f\left(x;\gamma ,\varepsilon \right)=\frac{1}{\pi \gamma \left[1+{\left(\frac{x-\varepsilon }{\gamma }\right)}^{2}\right]},\;\;x\in R,\;\gamma >0,\;\varepsilon \in R,$$

where γ and ε are called the scale parameter and the location parameter, respectively.

In other words, we say that a (univariate) random variable *X* has a Cauchy distribution with the parameters γ and ε (which we symbolically denote as X∼Cauchy(γ,ε)) if its probability density function is given by the formula in (24).

If γ=1 and ε=0, then the Cauchy distribution is called the standard Cauchy distribution. In this case, the probability density function from (24) reduces to the following:

(25) $$f\left(x\right)=\frac{1}{\pi \left(1+x^{2}\right)}.$$

### 5.3. Exponential Distribution

We will also take into account the situation when explanatory variables have an exponential distribution. This is a one-parameter continuous distribution with the following density function:

(26) $$f\left(x;\lambda \right)=\left\{\begin{array}{ll} \lambda \cdot e^{-\lambda x} & \mathrm{for}x\geq 0,\\ 0 & \mathrm{for}x<0, \end{array}\right.$$

where λ>0 is the rate parameter.

In other words, we say that a (univariate) random variable *X* has an exponential distribution with parameters λ (which we symbolically denote as X∼Exp(λ)) if its probability density function is as shown in (26).

### 5.4. Uniform Distribution

We will also consider a continuous uniform distribution in our empirical study. We say that a random variable *X* has a continuous uniform distribution on an interval [a;b] if its probability density function is determined as follows:

(27) $$f\left(x;a,b\right)=\left\{\begin{array}{ll} \frac{1}{b-a}, & x\in \left[a;b\right],\\ 0, & \mathrm{otherwise}, \end{array}\right.$$

where the bounds a,b, of an interval [a;b], are called the parameters of the corresponding uniform distribution.

In other words, we say that a (univariate) random variable *X* has a uniform distribution with parameters a<b (which we symbolically denote as X∼U([a;b])) if its probability density function is defined as shown in (27).

### 5.5. Beta Distribution

In our research, we will also assume that explanatory variables in the corresponding models are distributed according to the beta distribution. This distribution is continuous, with a density function expressed as follows:

(28) $$f\left(x;\alpha ,\beta \right)=\frac{1}{B(\alpha ,\beta )}x^{\alpha -1}{\left(1-x\right)}^{\beta -1},\;\;x\in \left[0;1]\;or\;(0;1\right),\;\alpha >0,\;\beta >0,$$

where α and β are the shape and scale parameters, respectively, and B(·,·) is the so-called beta function, determined as follows:   

(29) $$B\left(\alpha ,\beta \right)=\int_{0}^{1}t^{\alpha -1}{\left(1-t\right)}^{\beta -1}\;dt.$$

In other words, we say that a (univariate) random variable *X* has a beta distribution with the parameters α and β (which we symbolically denote as X∼Beta(α,β)) if its probability density function is defined as shown in (28).

In our investigations, we will also consider the right-skewed version of the beta distribution, denoted as Beta2.

## 6. Simulation Study

### 6.1. Nature of the Applied Explanatory Variables and Implemented Impurity Measures

All of the simulations presented in this section have been conducted using the R package [45]. The simulation code can be found in the GitHub repository (https://github.com/KrzyGajow/entropyMeasures/blob/main/Entropy.R, accessed on 14 October 2024). For proper use, the authors’ own package (see [15,16,17]), available at https://github.com/KrzyGajow/ImbTreeEntropy (accessed on 14 October 2024) needs to be installed, which is possible by entering the following commands (see Listing 1):
**Listing 1.** Necessary code for the *ImbTreeEntropy* package installation.
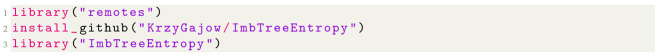



**Figure A1:**
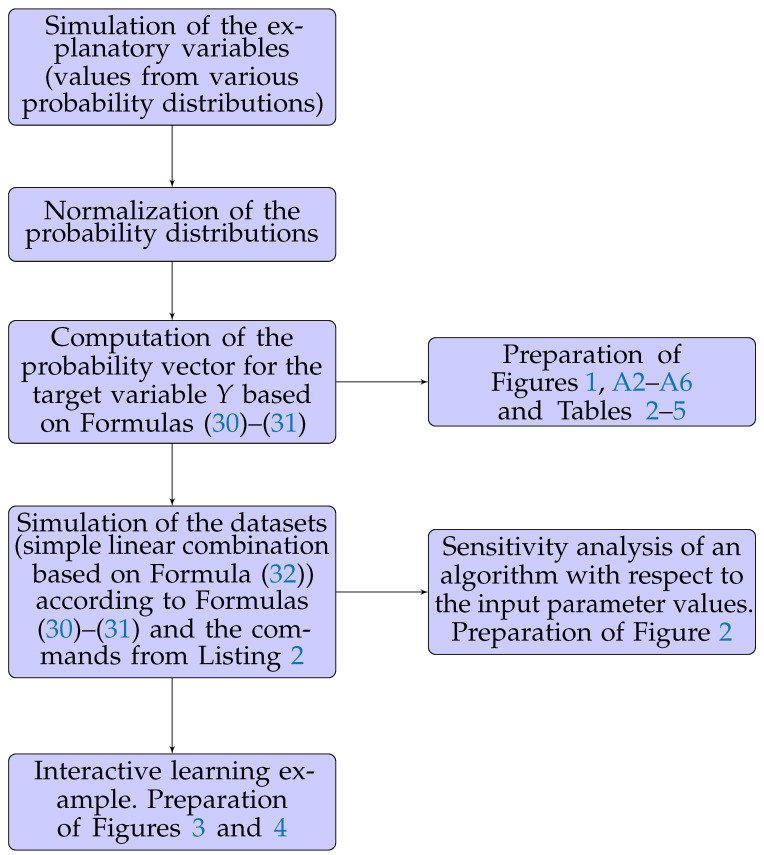
Schematic representation of the research procedure.

To understand the main steps of the analysis and the experiment structure, see Figure A1 in Appendix A.

**Figure 1:**
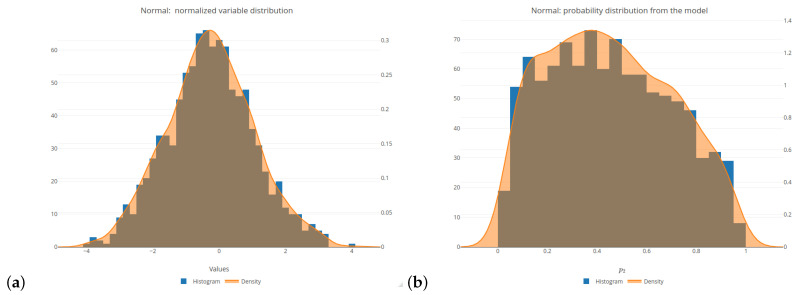

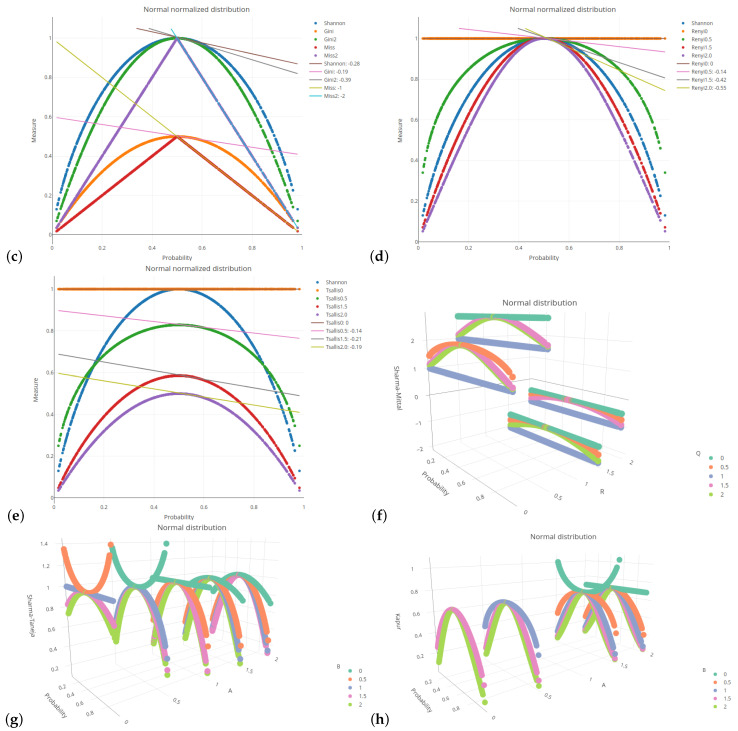
Characteristics and entropies of the normal distribution: (**a**) variable histogram; (**b**) $p_{2}$ histogram (probability from the Formula (31)); (**c**) Shannon; (**d**) Rényi; (**e**) Tsallis; (**f**) Sharma–Mittal; (**g**) Sharma–Taneja; (**h**) Kapur.

**Figure A2:**
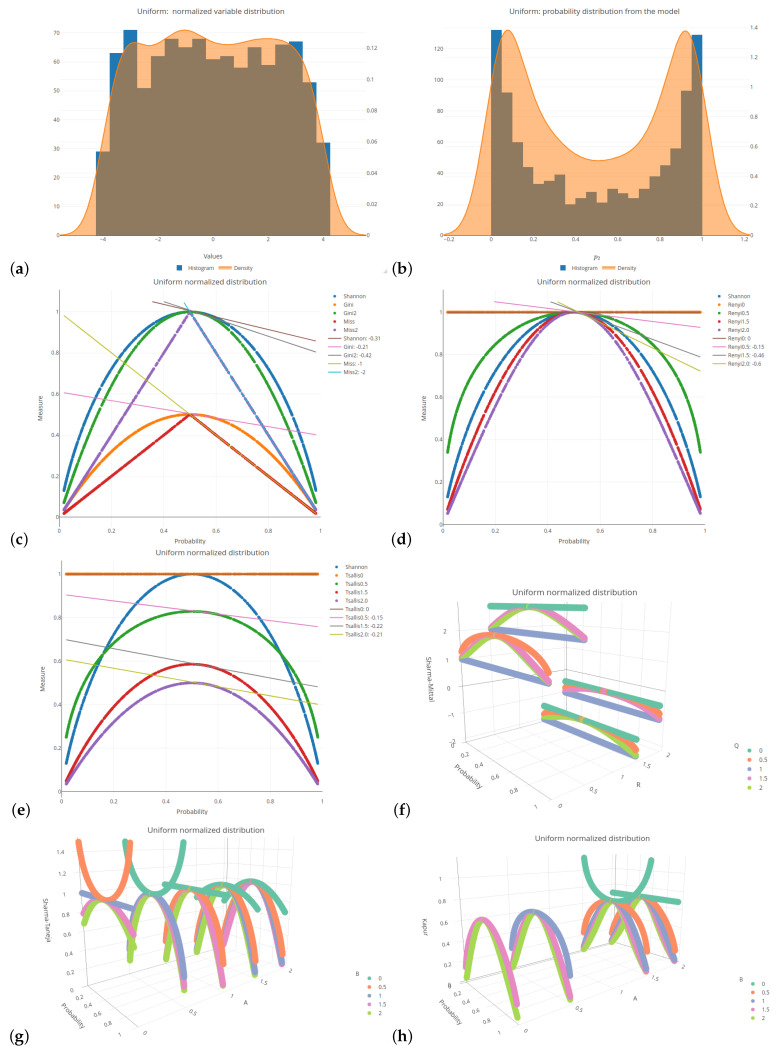
Characteristics and entropies of the uniform distribution. (**a**) Variable histogram; (**b**) $p_{2}$ histogram (probability from Formula (31)); (**c**) Shannon; (**d**) Rényi; (**e**) Tsallis; (**f**) Sharma–Mittal; (**g**) Sharma–Taneja; (**h**) Kapur.

**Figure A3:**
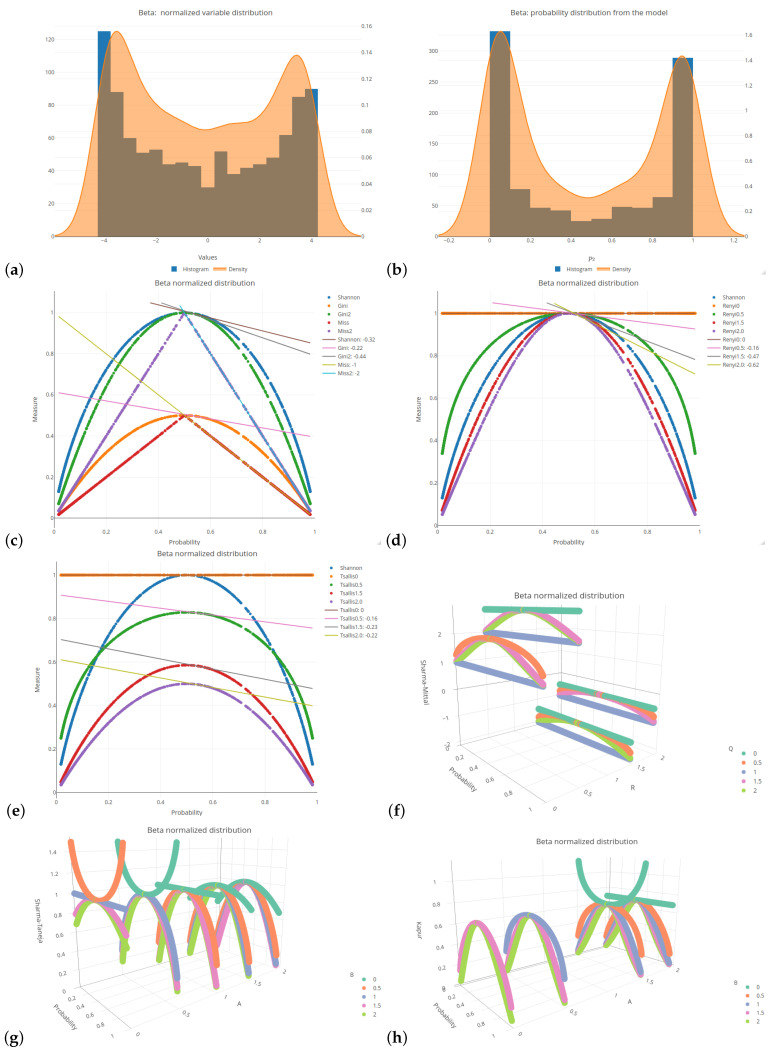
Characteristics and entropies of the beta distribution. (**a**) Variable histogram; (**b**) $p_{2}$ histogram (probability from Formula (31)); (**c**) Shannon; (**d**) Rényi; (**e**) Tsallis; (**f**) Sharma–Mittal; (**g**) Sharma–Taneja; (**h**) Kapur.

**Figure A4:**
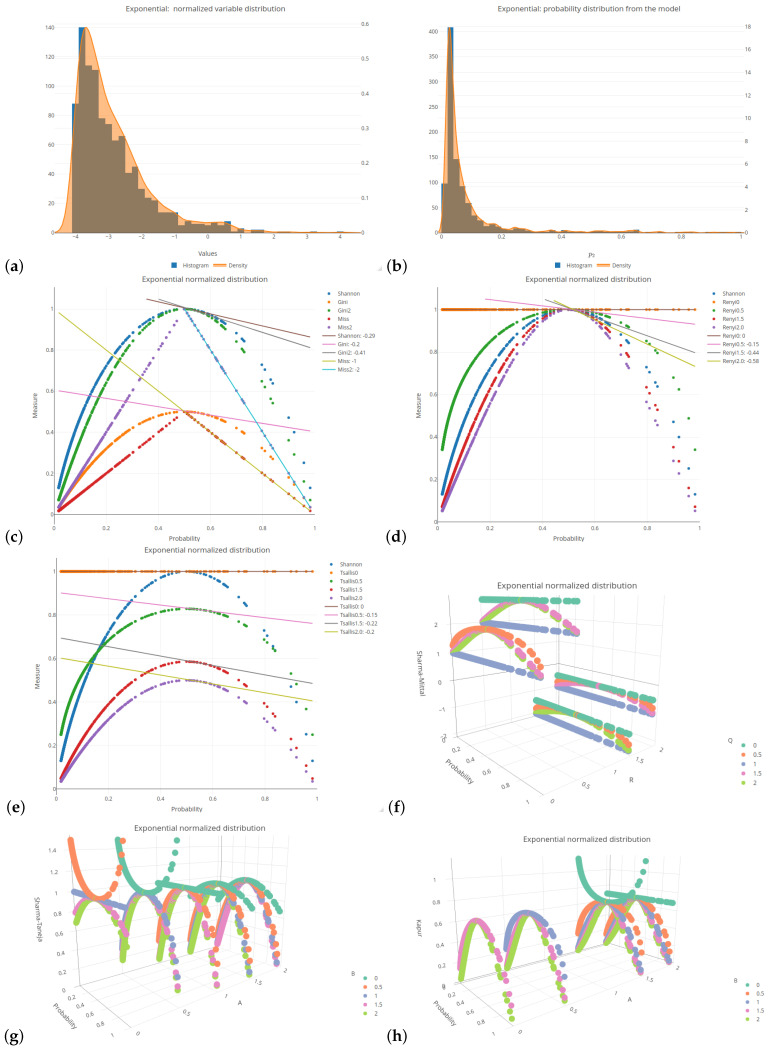
Characteristics and entropies of the exponential distribution. (**a**) Variable histogram; (**b**) $p_{2}$ histogram (probability from Formula (31)); (**c**) Shannon; (**d**) Rényi; (**e**) Tsallis; (**f**) Sharma–Mittal; (**g**) Sharma–Taneja; (**h**) Kapur.

**Figure A5:**
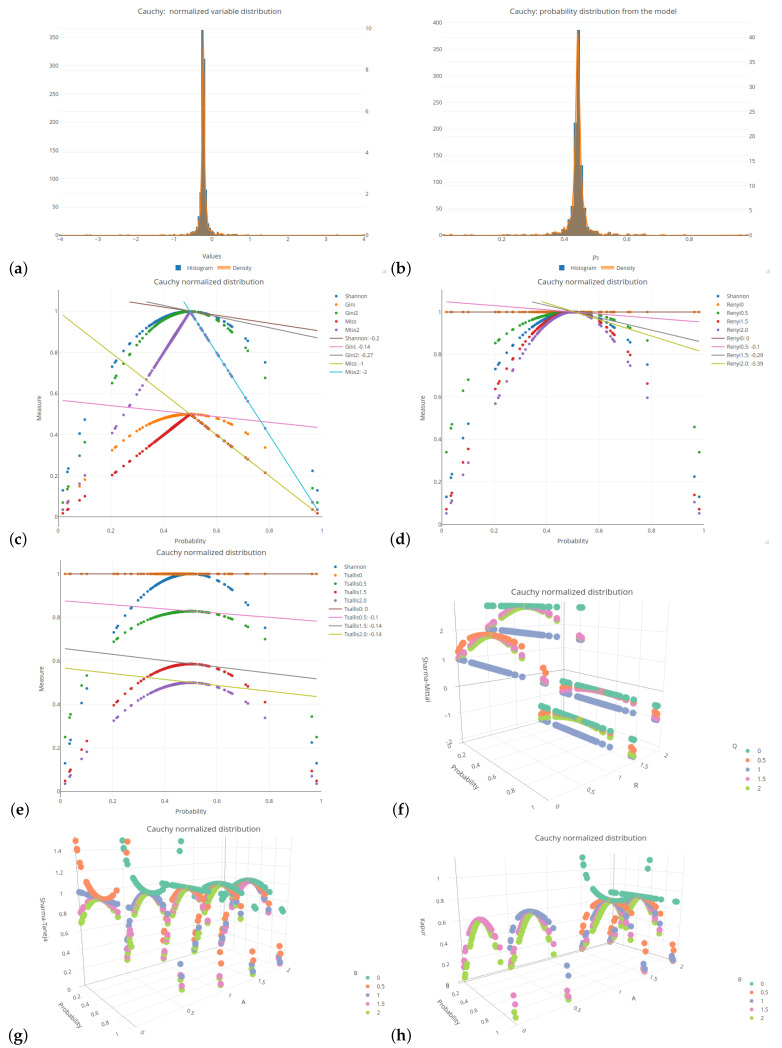
Characteristics and entropies of the Cauchy distribution. (**a**) Variable histogram; (**b**) $p_{2}$ histogram (probability from Formula (31)); (**c**) Shannon; (**d**) Rényi; (**e**) Tsallis; (**f**) Sharma–Mittal; (**g**) Sharma–Taneja; (**h**) Kapur.

**Figure A6:**
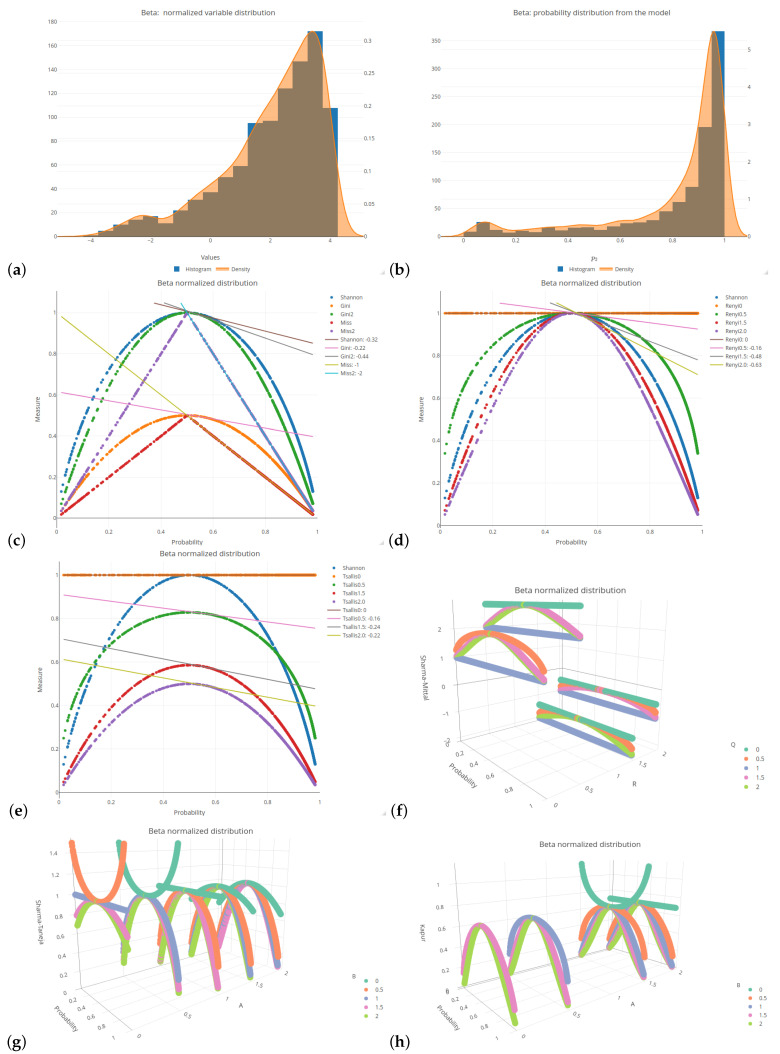
Characteristics and entropies of the Beta2 distribution. (**a**) Variable histogram; (**b**) $p_{2}$ histogram (probability from Formula (31)); (**c**) Shannon; (**d**) Rényi; (**e**) Tsallis; (**f**) Sharma–Mittal; (**g**) Sharma–Taneja; (**h**) Kapur.

We have started our empirical study by simulating the explanatory variables values (for the future regression logistic model) from various probability distributions in order to ensure a wide range of variability (see [46]). The following six distributions (with parameters specified in parentheses) have been selected: normal (rnorm function with a mean of 0 and a standard deviation of 1), uniform (runif function with an interval of [0;1]), beta (rbeta function with both the shape and scale parameters set to 0.5), exponential (rexp function with a parameter of 1), Cauchy (rcauchy function with the location and scale parameters set to 0 and 1, respectively), right-skewed beta—denoted as Beta2 (rbeta function with the shape and scale parameters set to 5 and 1, respectively). For more insight into these simulations, see the subplots (a) in Figure 1 and Figure A2, Figure A3, Figure A4, Figure A5 and Figure A6. In order to conduct comparisons between the simulated values, these values have been normalized (see [47] for comparison).

In the next step of our empirical study, we computed a probability vector for a target variable Y∈{1,2}. For that purpose, the parametric logistic regression model was used. Thus, the binary responses were obtained from the model of the following form:

(30) logit(p)=ln(p/(1−p))=Xβ,

where β is a vector of the model parameters (weights), and X stands for a feature matrix. In order to perform simulations using a given model based on an observable matrix X, we computed the dot product Xβ (value of the corresponding linear combination) by applying the inverse *logit* function:

(31) $$logit^{-1}=Prob\left\{Y=2|X\right\}=p_{2}=\frac{1}{1+\exp(-X\beta )}.$$

As an outcome, we obtain the probability that a given observation belongs to the class of a target variable *Y* labeled as 2. Consequently, a response (target) variable *Y* is a Bernoulli random variable with parameter $p_{2}$. This variable returns label 1 if $p_{2}\leq 0.5$ or label 2 if $p_{2}>0.5$. In the corresponding code below, the text labels _0_ and _1_ are used for return labels 1 and 2, respectively.

A linear combination Xβ refers to the values simulated from the considered (normalized) probability distributions. Subplots (b) of Figure 1 and Figure A2, Figure A3, Figure A4, Figure A5 and Figure A6 depict the distributions of a response variable *Y* in the logistic model generated for the selected probability distributions of the explanatory variables. Due to the fact that some of these distributions generate only positive values by default, these variables are normalized as described above, because both positive and negative input values are needed in order to obtain the probability values both from the areas above and below the threshold of 0.5.

All of the six distributions, selected as the probability distributions of the explanatory variables in our logistic regression model, are presented in the same format (see Figure 1 and Figure A2, Figure A3, Figure A4, Figure A5 and Figure A6). Subplots (a) and (b) of these figures depict a histogram related to the selected distributions of the explanatory variables values and a histogram of the probabilities related to a response (target) variable of the specified logistic model, respectively. Subplots (c)–(h) of the mentioned figures present the values of various impurity (entropy) measures obtained for different sets of parameters. They include the Shannon, Gini, and missclassification (denoted as Miss) measures. In order to compare the Gini and Miss measures with the Shannon measure, the first two measures were properly rescaled (multiplied by 2). The following combinations of parameters were used for the considered entropy measures: q∈{0.0,0.5,1.5,2.0} for the Rényi and Tsallis entropies, q∈{0.0,0.5,1.0,1.5,2.0} and r∈{0.0,0.5,1.0,1.5,2.0} for the Sharma–Mittal entropy, and α∈{0.0,0.5,1.0,1.5,2.0} and β∈{0.0,0.5,1.0,1.5,2.0} for the Sharma–Taneja and Kapur entropies. Note that, due to the conditions imposed on the parameters in the formulas for entropy measures, certain combinations are not allowed. Consequently, they are not displayed in the graphs.

The 2D graphs, showing the values of the selected impurity measures, are included with the added tangent lines at the point of 0.55 (see also Table 2). They illustrate the rate of decline (the regression slope) in the values of the considered measures in the tied or ambiguous situations. This involved estimation of the linear regression for the probability values ranging from 0.5 to 0.6. In the case of two-parameter measures, the corresponding slopes are collected in Table 3, Table 4 and Table 5.

Let us consider the most popular probability distribution—the normal distribution. This case is presented in Figure 1 and Table 2. The Shannon entropy is depicted in three subfigures, since it is the impurity measure that is commonly present in most of the available software. Figure 1c shows a faster decline in the Shannon entropy values compared to the Gini measure in the middle part of the graph. This is further indicated by the comparison of the slope values relating to the corresponding lines ((−0.29) compared to (−0.19)). The Rényi entropy has a greater slope if the parameter *q* increases (see Figure 1d); after exceeding a value of 1, it is greater than in the Shannon entropy case. In the case of the Tsallis entropy, as shown in Figure 1e, there is no clear upward or downward trend if we change the value of parameter *q*, but the slope of the curve is always smaller than for the Shannon entropy. The Rényi entropy expands and contracts sideways with respect to the Shannon entropy, always maintaining a maximum value of 1, whereas the Tsallis entropy fluctuates in comparison to the Shannon entropy, trough decreasing and increasing its maximum value.

In order to improve visual clarity, the regression curves have not been placed in Figure 1f–h. Instead of depicting them, slopes of the regression curves are given in Table 3, Table 4 and Table 5. The minus sign indicates a situation where the measure cannot be calculated for a given combination of parameters due to the constraints on the parameter values in Formulas (15)–(19). In order to highlight the relationships between the slopes, we introduced the color saturation for individual values. The green color illustrates the highest values, while the red one shows the lowest values. It is important to notice that the terms ‘highest/lowest values’ should be understood as a distance from 0 but not as a tendency towards verticality. No matter what the value of parameter *r* is set to in the Sharma–Mittal entropy, setting 1 or 2 as the values of parameter *q* will result in constant measurement values (see teal blue and royal blue colors in Figure 1f). This combination cannot be used to train a tree. Changing the parameter values will affect the range of values of this measure, from (−2) to 3 on the vertical axis. The decision tree learning algorithm aims to find a partition that minimizes the impurity as much as possible. This is particularly important at the edges of the graph where the probability values are 0 and 1. Consequently, there are certain combinations of parameters for the Sharma–Taneja entropy that should be avoided, even though the theory does not explicitly prohibit them. This is depicted by the two parabolas in the top-left corner of Figure 1g, with values that increase as the classification quality improves. A similar trend can be observed in the last Figure 1h, illustrating the Kapur entropy. When looking at the 3D graphs related to the last three entropies, it is evident that if parameter *q* or parameter β increase, then the corresponding curves are pulled inward, which results in steeper tangents. The graphs for the remaining five distributions can be found in Appendix A.

Let us now delve into a more detailed discussion of the results presented in Table 2, Table 3, Table 4 and Table 5. In Table 2, the extreme values of 0 and ≤−1 have been excluded from the color scale in order to better capture small differences between the slope values. In the columns, the distributions of the variables are arranged based on the observed trend in the slope coefficient from the highest to the lowest values. The Cauchy distribution generates the most horizontal regression line with the largest slope, creating a greater distance between its values and the other distributions, compared to the other distributions. On the right-hand side of Table 2, there are two beta distributions (one two-modal and one right-skewed) that produce the most vertical slope angles. Regardless of the impurity measure (and its parameter), the minimum value is approximately 62% of the maximum value, e.g., for the Shannon entropy, it equals (−0.20)/(−0.32)=62.5%. The distribution ordering (in rows) remains the same as before for the other three tables (Table 3, Table 4 and Table 5), i.e., we observe the same dependencies. For the Sharma–Mittal entropy, the smallest values are grouped in the upper-right corner of Table 3. The slope increases if the parameter *r* decreases and the parameter *q* increases. For the Sharma–Taneja entropy, the symmetry of slope with respect to the diagonal is seen in Table 4—the slope value decreases if both parameters increase. A similar trend to that of the Sharma–Taneja measure is observed in the case of the Kapur entropy (see Table 5).

### 6.2. Sensitivity Analysis of Entropy Parameters

In order to simulate the datasets according to the formulas in (30) and (31) and the commands from Listing 2, the following simple linear combination was created avoid increasing or decreasing impacts of a single variable:

(32) $$linearCombination=\sum_{l=1}^{p}x_{l}^{T},$$

where, for a given *l*, $x_{l}^{T}$ is an empirical realization (observation) of a feature $X_{l}$ from a random vector space $X^{p}$ for a given probability distribution. The same weight 1 is assigned to each variable, i.e., the regression coefficient for each variable is determined as 1. The formed linear combination, which is the input of our model, resulted in the selection of 31% observations with a label of _0_ and 69% observations with a label of _1_ (see Listing 2).

**Listing 2.** Code for an assignment of the return labels of a target variable *Y*.

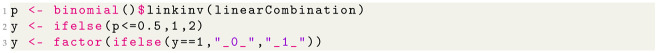



In order to examine the behavior of the obtained results, we conducted a sensitivity analysis of an algorithm with respect to the input parameter values. For each algorithm combination run, we considered different values of the input parameters. The following combinations of the entropy parameters were used: for the Rényi and Tsallis entropies, *q* ranged from 0 to 5, with a step of 0.5; for the Sharma–Mittal entropy, *q* and *r* ranged from 0 to 5, with a step of 0.5; and for the Sharma–Taneja and Kapur entropies, α and β ranged from 0 to 5, with a step of 0.5. As mentioned earlier, some parameter combinations were not allowed. The hyper-parameters for an algorithm were set as follows: the minimum number of observations that must exist in a leaf Min_obs∈{10,50,100}, i.e., 1%, 5%, and 10% of the observations in the dataset; the depth of the tree depth∈{5,6,7,8,9,10}. The overfitting method leafcut was set as in [15,17]. The simulation was performed in a five-fold validation regime with a predefined seed of the random number generator. According to the ratio (1−1/k)%−(1/k)%, where *k* is the number of folds, each training sample consisted of 80% of the total number of observations, while the remaining 20% formed the validation sample.

The number of different combinations of input parameters is 5796. Each combination was additionally checked on five cross-validation partitions, which gives a total of 28,980 algorithm runs.

**Figure 2:**
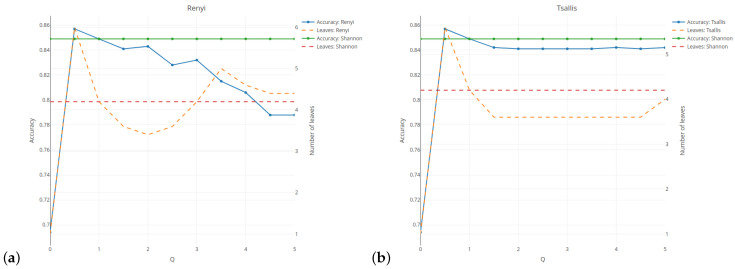

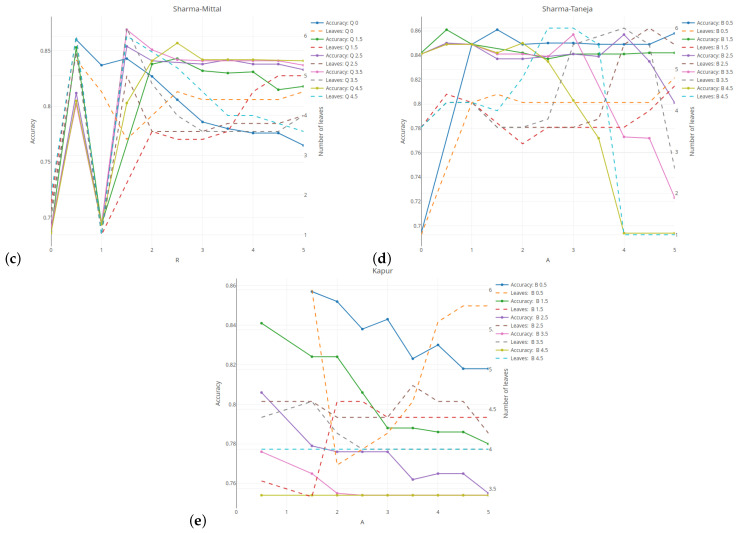
Analysis of the results quality and complexity of the tree for five different entropies: (**a**) Rényi, (**b**) Tsallis, (**c**) Sharma–Mittal, (**d**) Sharma–Taneja, (**e**) Kapur.

In order to ensure the consistency and clarity of our work’s argumentation, we have included partial results based on the fixed input hyper-parameters of the algorithm. The detailed results can be replicated and viewed using the provided source code (https://github.com/KrzyGajow/entropyMeasures/blob/main/Entropy.R, accessed on 14 October 2024). Figure 2 illustrates the relationship between the classification quality (on the left vertical axis) and the tree complexity measured by the number of leaves (on the right vertical axis) for five selected entropies. Here, the results are given for the minimum number of observations set as 5% and the tree depth set as 5. The results are averaged over five cross-validation runs of the validation sample. For the Rényi and Tsallis entropies (see Figure 2a,b), the values of the parameter *q* are given on the horizontal axis. The results related to the classification quality are presented in the form of a solid line, while the numbers of leaves in the tree are presented as a dashed line.

For comparative purposes, the green and red horizontal lines indicate the results for the case when the Shannon entropy has been taken into account. The average quality classification for the Shannon entropy is lower than 85% for a tree with an average of 4.2 leaves. In Section 6.1, we discovered the inward shrinking and expanding properties of the Rényi entropy. This behavior strongly affects the shape of the measure with respect to the probability, which is reflected in the the regression slope. As shown in Figure 2a, this greatly influences the results. With an increase in the parameter *q*, the tree loses its predictive power (see blue line). The complexity of the tree follows a U-shaped pattern. For *q* set at 2, the tree has almost the same classification quality as the Shannon entropy, but the number of leaves is on average 0.7 smaller. When using the Tsallis entropy, the shape is slightly distorted, but only the scale of values changes. This leads to a slightly lower classification quality of 85%, with a tree that has on average 0.5 fewer leaves. The results are consistent and are not highly dependent on the parameter *q* when it is above 1.5. For both entropies, a better classification quality than for the Shannon entropy is obtained for *q* = 0.5, but the complexity of a tree is higher.

The Sharma–Mittal entropy-based results (see Figure 2c) show two cases when the tree does not divide the root at all (*r* = 0 or 1). The best classification—of above 87%—is observed for *r* = 1.5 and *q* = 3.5 (see violet–red line). However, it is obtained at the expense of the tree’s size. The optimal balance between the quality and the tree size is attained for r≥3.5 and *q* = 4.5, which results in an almost 85% accuracy (see spring-green line) with four or fewer leaves (see cyan dashed line). As shown in subfigure Figure 2d, using the Sharma–Taneja entropy with parameter β set as 0 (see yellow–orange dashed line) or above 3.5 (see cyan dashed line) does not form a tree. Setting parameter α as greater than or equal to 1 and parameter β as 0.5 yields results that are similar to those for the Shannon entropy in both the classification quality and the number of tree leaves. This is due to the fact that if we examine the relevant part of Figure 2g (see yellow–orange dots) and Figure 2c (see royal blue dots), we observe that both curves share similar properties. Simultaneous increases in both parameters lead to a deterioration of the classification quality and the tree’s growth. Due to the restricted parameter combinations, some curves start in the middle of Figure 2e. A decrease in the classification quality is observed for each value of parameter β if the values of parameter α increase.

**Figure A7:**
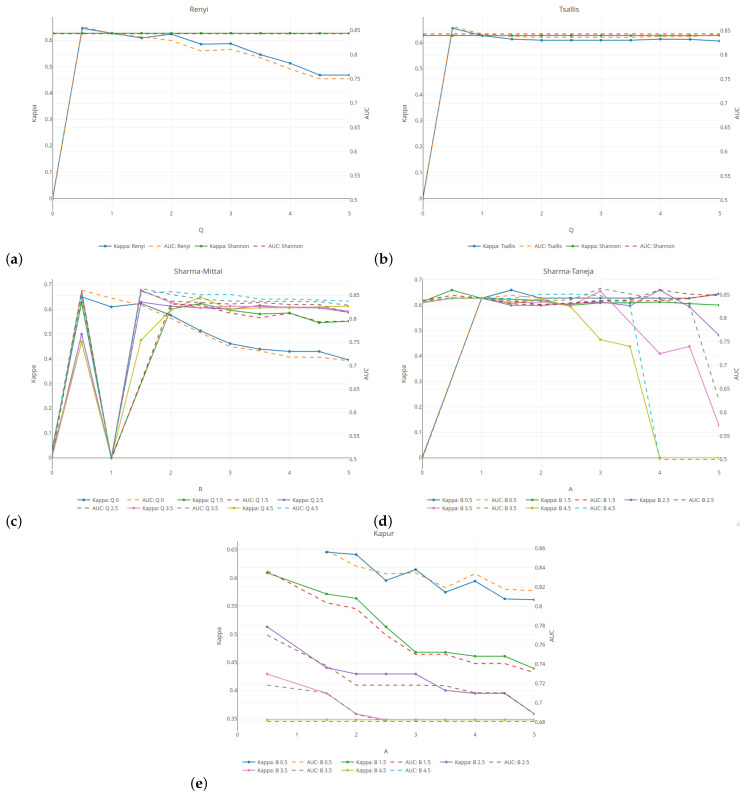
Analysis of the Kappa and AUC measures for five different entropies: (**a**) Rényi, (**b**) Tsallis, (**c**) Sharma–Mittal, (**d**) Sharma–Taneja, (**e**) Kapur.

The remaining results showing the Kappa and AUC measures used for unbalanced data sets are shown in Figure A7 from Appendix A.

### 6.3. Interactive Learning

In the current section, we will present the interactive learning procedure only for the Shannon entropy and avoid considering the other entropy measures, since adding the other cases would unnecessarily increase the size and the complexity of our paper.

**Figure 3:**
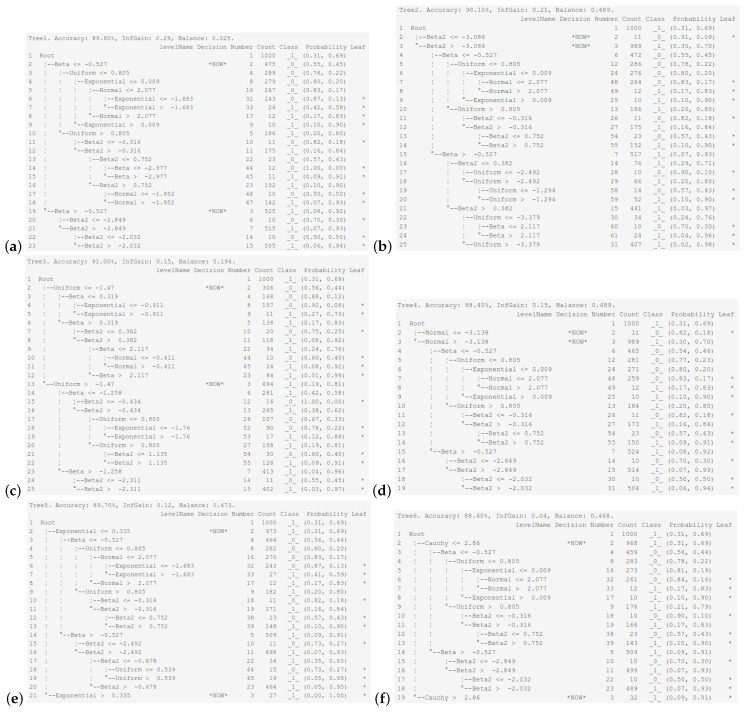
The structure of classification trees in interactive learning during the first decision. Subplots (**a**–**f**) presents six trees for different variables at the first level of division. The asterisk indicates a terminal node.

Figure 3 displays the final tree structure obtained in the interactive learning mode after selecting one of the six explanatory variables for the initial root split. The tree was built using the default hyper-parameter settings. The maximum tree depth is 5, and the minimum number of observations in a leaf is 10. The Shannon entropy is used as the impurity measure, the best split is chosen at the attribute level, and the ambiguity threshold is set at 1, which means that a decision will always be made by an expert. The decision column following the tree structure indicates where an expert’s decision is made, as indicated by the text *NOW*. The most effective discriminating explanatory variable is the beta distribution, with an information gain of 0.29 and a classification accuracy of 89.90%. Figure 3a depicts the case of the beta explanatory variable, also showing the theoretical tree trained entirely in an automatic mode. The next explanatory variables in the ranking, made with respect to the discriminatory power (at the root division level), are the Beta2, uniformly, normally, exponentially, and Cauchy-distributed random variables. This order is largely consistent with the regression slope decline rates ranking discussed earlier. This good discriminatory power of the beta-distributed explanatory variable and the tendency towards the construction of a balanced tree result from the U-shaped nature of the beta distribution, which pushes objects from two classes to the opposite distribution tails. Returning to the dependencies in Table 2, the slope of the beta-distributed variable for the Shannon entropy is the largest, which means that this variable separates the two populations in the fastest possible way.

Our study shows that the probability distributions of the explanatory variables for which the best balanced trees are obtained are the beta and uniform distributions. That is because, based on the histograms of the probability distributions (responses presented in subplots (b)), both of the mentioned distributions are U-shaped bimodal distributions, whereas imposing that explanatory variables have some of the remaining probability distributions leads to the construction of chain-like trees with numerous small terminal nodes.

**Figure 4:**
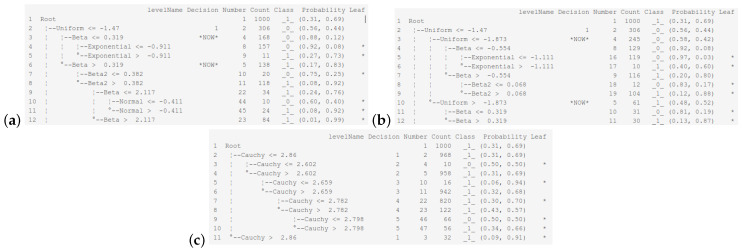
Different tree structures obtained in interactive learning mode by selecting the intervening variables on the first and second partition: (**a**) normal and beta, (**b**) uniform and uniform, (**c**) Cauchy and Cauchy. The asterisk indicates a terminal node.

Let us analyze the tree structure obtained by choosing the uniform distribution as the explanatory variable distribution used in the first tree’s partition. In the first node (see the upper part of Figure 3c), we have almost a tie situation regarding the probability vector for both classes. The second node overrepresents class _1_, since it contains 81% of the observations from that class. While splitting a node with the condition ≤ at the next sublevel, the best variable turns out to be of the beta distribution, which is followed by the cases when the best variable has the exponential, Beta2, normal, Cauchy, and uniform distributions. Assuming that the first and last distributions from the corresponding ranking are the distributions of variables used in the tree-building process, we obtain the most balanced trees, as depicted in Figure 4a,b.

In turn, if we consider a variable with the Cauchy distribution in the first tree’s partition, then the second partition has the same order of potential subsequent partitions as in the root. While selecting the worst variable for splitting until the very end of the tree training, we observe that it is always the same variable with the Cauchy distribution. The final tree structure is a chain with single small branches, which are leaves (without perfect classifications) coming from the main subtree, as shown in Figure 4c. The quality of the obtained tree is only 69%. It seems as if the root had not been divided at all, and all observations were assigned to class _1_.

## 7. Summary

### 7.1. Stated Goals

The main goal of our study was to determine the impact that the selection of the explanatory (input) random variable distribution might have on the values of the considered entropy measures, given that the feature vector was governed by the logistic regression model. We assumed that our explanatory variables were distributed according to the normal, Cauchy, uniform, exponential, beta and Beta2 distributions. In addition, six entropy measures, namely the Shannon, Rényi, Tsallis, Sharma–Mittal, Sharma–Taneja, and Kapur entropies were taken into account in our research. The performed analysis used the mentioned measures in the interactive learning of decision trees, particularly in tie-breaking situations where an expert’s final decision was expected to be made. In our empirical study, we simulated the values of the explanatory variables using various settings of probability distributions in order to include a possibly wide range of variable diversities.

### 7.2. Conclusions and Discussion

The following conclusions can be drawn based on the conducted research. The introduced algorithm shows the best solutions—either with respect to the decrease in the impurity measures values, or with respect to the assumed distributions of explanatory variables, or—going further—with respect to the possibility of the selection of different division points for the same variable. The presented analysis shows how the algorithm should be tuned with respect to the hyper-parameters in order to provide the optimal flexibility for an expert, e.g., in the form of delivered information regarding the extent to which the homogeneity degree must be reduced to make a division possible. This reduction is different depending on the chosen entropy measure. If the tree-pruning or the variable selection criteria are based only on the value of the information gain, some variables will not be selected for learning due to the different entropy values for different probability distributions of the input data. Our study indicates which probability distribution will be appropriate for the considered impurity measure during interactive learning. It also shows which parameter values are not allowed, since for ideal classification, the entropy measures should decrease and not increase. In some cases, we have constant values of the measures that are independent of probability, such as the Rényi and Tsallis entropies, with parameter *q* set at 0. Some distributions are more non-linear and sensitive to changes in probability, and some distributions do not generate probability values within a certain interval. For example, the Cauchy distribution generates values more frequently in the middle and less frequently at the tails, whereas the exponential distribution generates samples less frequently at a value of 0.5. By analyzing the assumed parameters of the considered entropy measures in the sensitivity analysis section, it can be concluded that the input parameters have a significant impact on the obtained results. It is possible to achieve superior results compared to those obtained using the commonly used the Shannon entropy by maintaining a simpler tree structure. This allows for better interpretability and reduces the need for an expert intervention in the learning process.

The conducted investigations are important for future developments in the area of Explainable Artificial Intelligence (XAI). This is essentially due to a strong need for clarity, explainability, and integrability of the AI systems. Thus, it is relevant to create the AI systems that are not only efficient but also ethical and easy to interpret and comprehend. We believe that our research meets these requirements, as it forms a bridge between the complexity and comprehensibility of these systems while providing useful and ethical tools that may be highly successful and extremely valuable in supporting decision-making processes from diverse sections of the economy and industry. Consequently, it can greatly help decision makers who are not necessarily familiar with the AI techniques but who are undisputed experts in their own areas of interest. In particular, the proposed implementation of interactive learning allows us to create new optimal tree splitting by taking into account expert tips and knowledge, which is particularly significant when tied or ambiguous situations occur (these kinds of situations are discussed in Section 2.5 and Section 6).

### 7.3. Validity Threats

In general, it can be stated that there are no threats related to the validity assessment of the results obtained in our research. This study was conducted based on numerical simulations performed using a generally available and free programming language, R. As previously mentioned, the source code of the implemented *ImbTreeEntropy* algorithm is also publicly available, in particular for validation and error reporting. No external data were used, as all simulations were carried out appropriately while preserving the seed for pseudo-random number generators, which enables each researcher to completely reproduce our empirical study. We aimed to employ a large spectrum of probability distributions for different sets of their characteristics. In order to capture the hidden dependencies, our results were presented in both tabular and visual versions. The entry barrier in understanding the essence of the proposed approach and the received results might be the level of the researcher’s own knowledge of computer technologies, statistical and machine learning methods, and their possessed proficiency in using a given programming language.

### 7.4. Significance of the Conducted Study

The proposed research is essential and vital for further developments and enhancements of the Explainable Artificial Intelligence (XAI) field of study [48,49]. The necessity of such developments and enhancements results from a permanently increasing need for transparency and accountability of the AI systems. As AI becomes more integrated with the decision-making processes from various branches of economy and industry, it is crucial to ensure that these systems are not only effective but also interpretable and understandable to human users. Our work responds to these expectations and goals by providing frameworks and methods that allow users to actively participate in the model’s learning and gain insight into how the AI models make their decisions. This, in turn, builds necessary trust, facilitates the creation of more informed decision-making processes and strategies, and helps to identify and mitigate biases. By bridging the gap between complex AI algorithms and human comprehension, this research supports the creation of ethical, reliable, and responsible AI systems.

One open problem is what applications of entropy measures should be considered in future research. Since many types of entropy functions have been constructed, improved, and developed throughout the years, the potential scope of their possible applications have rapidly expanded in recent decades. This range of applicability encompasses not only the well-established classical entropies and their generalizations, such as the Shannon, Rényi, Tsallis, Sharma-Mittal, Sharma-Taneja, and Kapur entropies, but also several relatively new entropies that have been proposed, e.g., entropy functions based on the concept of fractional calculus, known as the fractional entropies (see Lopes and Machado [50] for a comprehensive overview regarding these entropy functions). This additionally comprises the classification methods using the uncertainty measures that have been introduced within the Dempster–Shafer evidence theory (see Peñafiel et al. [36], Urbani et al. [51], Ubriaco [28], and Balakrishnan et al. [52] for details). As summarized in Lopes and Machado [25], Peñafiel et al. [36] and Mageed and Zhang [53], the fields of knowledge where entropy and uncertainty measures may be practically applied in the near future, include stochastic systems, fault detection, image processing, fractal theory, visualization systems, financial and commercial strategies, medicine and healthcare, queuing theory, engineering systems, statistical mechanics, and chromosome and DNA analysis (see, e.g., Figure 4 in Lopes and Machado [24], where a map of possible applications of fractional entropies is depicted). In particular, it involves the development of expert and recommendation systems, which codify the acquired skills and knowledge of specialists in the clusters of interpretable rules and recommendations. This later supports the decision makers in their everyday work. This may become very relevant in the healthcare sector, where it should be vital to know why the proposed treatment of a certain illness is recommended, or in financial systems, where it may be required to demonstrate that the rejection of a credit application is not connected with discriminatory reasons such as gender or race.

In our future studies, we aim to investigate how newly created impurity measures—obtained by taking the weighted combination or aggregation of the previously considered entropy measures—may behave. Furthermore, the use of fractional entropies and Dempster–Shafer evidence theory for the DT learning may also be worth considering in our further research.

## Figures and Tables

**Table 1:** Overview of the selected DT induction algorithms.

Algorithm	Rule (Criterion)	Advantages	Disadvantages
CART	Gini index-based information gain	A versatile algorithm that has the ability to generate both classification and regression trees	Can overfit, requires rigorous parameter tuning and careful pruning; is biased towards selecting split variables
ID3	Entropy-based information gain	Simple, easy to understand, implement, and interpret	Prone to overfitting, does not handle numeric attributes or missing values
C4.5	Gain ratio	Handles both categorical and continuous attributes, reduces overfitting by conducting appropriate pruning methods	Works slower for larger datasets
C5.0	Gain ratio	Efficient for large data, creates smaller trees	Can overfit if no proper pruning is carried out
CHAID	Pearson’s chi-squared test (if a target variable is categorical), F-test (if a target variable is continuous)	Can handle missing values; good for analyzing interactions in the categorical data	Allows for categorical input (predictor) variables only; can produce large trees and is sensitive to data changes

**Table 2:** Regression slopes for the probabilities between 0.5 and 0.6 (with relation to the considered impurity measures).

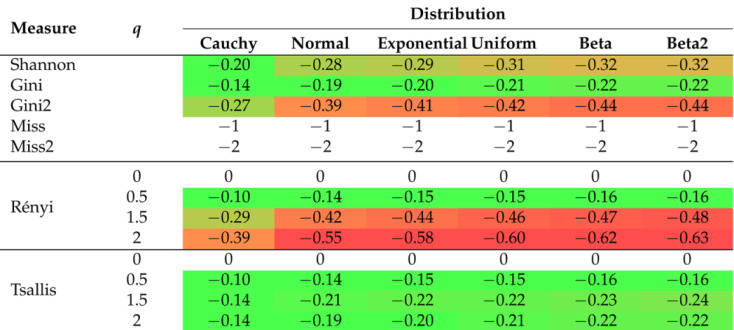

The color scale allows for better identification of individual values, where red indicates the lowest values and green indicates the highest values.

**Table 3:** Regression slopes for the probabilities between 0.5 and 0.6 (the Sharma–Mittal entropy case).

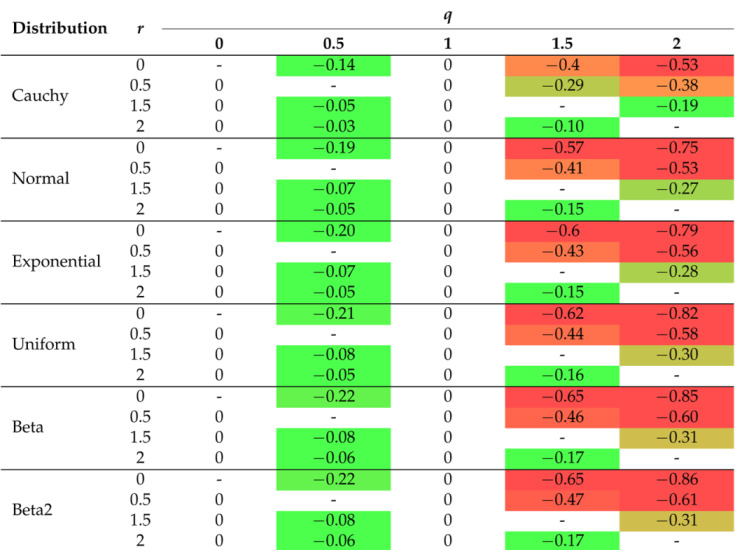

The color scale allows for better identification of individual values, where red indicates the lowest values and green indicates the highest values.

**Table 4:** Regression slopes for the probabilities between 0.5 and 0.6 (the Sharma–Taneja entropy case).

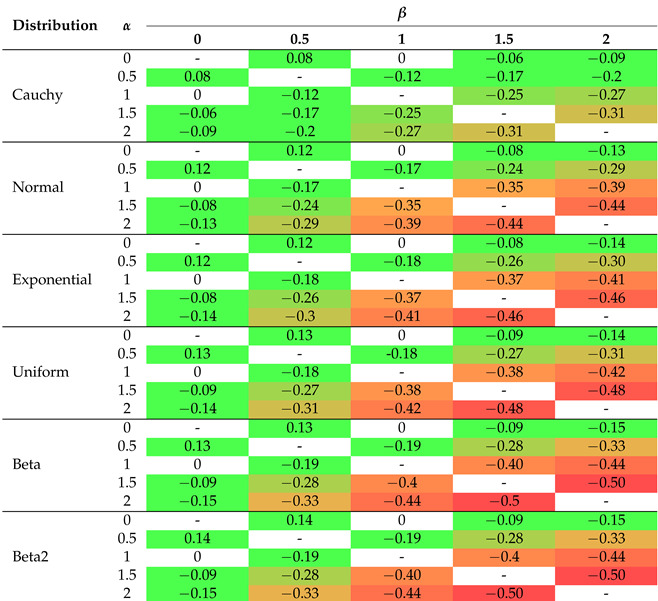

The color scale allows for better identification of individual values, where red indicates the lowest values and green indicates the highest values.

**Table 5:** Regression slopes for the probabilities between 0.5 and 0.6 (the Kapur entropy case).

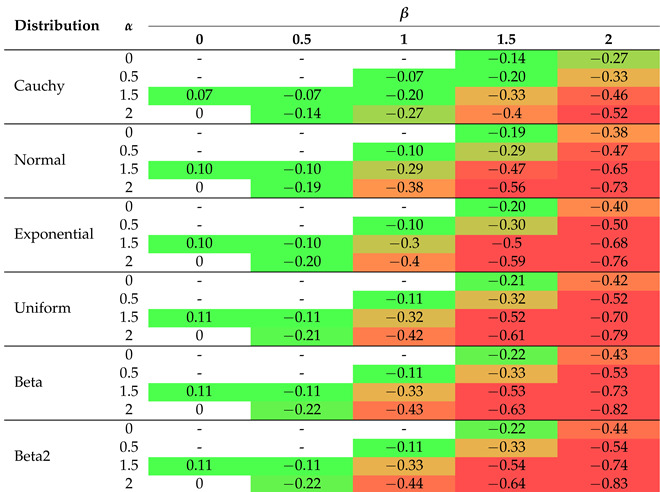

The color scale allows for better identification of individual values, where red indicates the lowest values and green indicates the highest values.

## Data Availability

The data presented in this study are openly available in Github repository. https://github.com/KrzyGajow/entropyMeasures/Entropy.R (accessed on 14 October 2024).

## References

[1] Clopper C.J., Pearson E.S. (1934). The Use of Confidence or Fiducial Limits Illustrated in the Case of the Binomial. Biometrika.

[2] Breiman L., Friedman J.H., Olshen R.A., Stone C.J. (2017). Classification and Regression Trees.

[3] Quinlan J.R. (1986). Induction of decision trees. Mach. Learn..

[4] Quinlan J.R. (1993). Pruning Decision Trees. C4.5.

[5] Quinlan J.R. (2004). Data Mining Tools See5 and C5.0. http://www.rulequest.com/see5-info.html.

[6] Hancock T., Jiang T., Li M., Tromp J. (1996). Lower Bounds on Learning Decision Lists and Trees. Inf. Comput..

[7] Quinlan J. (1987). Simplifying decision trees. Int. J. Man-Mach. Stud..

[8] Mingers J. (1989). An empirical comparison of selection measures for decision-tree induction. Mach. Learn..

[9] Esposito F., Malerba D., Semeraro G., Kay J. (1997). A comparative analysis of methods for pruning decision trees. IEEE Trans. Pattern Anal. Mach. Intell..

[10] Ankerst M., Ester M., Kriegel H.P. Towards an effective cooperation of the user and the computer for classification. Proceedings of the Sixth ACM SIGKDD International Conference on Knowledge Discovery and Data Mining.

[11] Liu Y., Salvendy G. (2007). Design and evaluation of visualization support to facilitate decision trees classification. Int. J. Hum.-Comput. Stud..

[12] van den Elzen S., van Wijk J.J. BaobabView: Interactive construction and analysis of decision trees. Proceedings of the 2011 IEEE Conference on Visual Analytics Science and Technology (VAST).

[13] Pauwels S., Moens S., Goethals B. (2014). Interactive and manual construction of classification trees. BeneLearn.

[14] Poulet F., Do T.N. (2008). Interactive Decision Tree Construction for Interval and Taxonomical Data. Visual Data Mining.

[15] Gajowniczek K., Ząbkowski T. (2021). ImbTreeEntropy and ImbTreeAUC: Novel R Packages for Decision Tree Learning on the Imbalanced Datasets. Electronics.

[16] Gajowniczek K., Ząbkowski T. (2021). Interactive Decision Tree Learning and Decision Rule Extraction Based on the ImbTreeEntropy and ImbTreeAUC Packages. Processes.

[17] Gajowniczek K., Ząbkowski T. (2021). ImbTreeEntropy: An R package for building entropy-based classification trees on imbalanced datasets. SoftwareX.

[18] Rokach L., Maimon O. (2005). Decision Trees. Data Mining and Knowledge Discovery Handbook.

[19] Kearns M., Mansour Y. On the boosting ability of top-down decision tree learning algorithms. Proceedings of the Twenty-Eighth Annual ACM Symposium on Theory of Computing—STOC ’96.

[20] Dietterich T., Kearns M., Mansour Y. Applying the weak learning framework to understand and improve C4.5. Proceedings of the ICML.

[21] Fayyad U.M., Irani K.B. The attribute selection problem in decision tree generation. Proceedings of the Tenth National Conference on Artificial Intelligence.

[22] (1977). Friedman. A Recursive Partitioning Decision Rule for Nonparametric Classification. IEEE Trans. Comput..

[23] Rounds E. (1980). A combined nonparametric approach to feature selection and binary decision tree design. Pattern Recognit..

[24] Lopes A.M., Tenreiro Machado J.A. (2019). Fractional-order modeling of electro-impedance spectroscopy information. Applications in Engineering, Life and Social Sciences, Part A.

[25] Lopes A.M., Machado J.T. (2013). Fractional order models of leaves. J. Vib. Control.

[26] Lopes A.M., Machado J.A.T. (2020). A Review of Fractional Order Entropies. Entropy.

[27] Akimoto M., Suzuki A. (2001). Proposition of a new class of entropy. J. Korean Phys. Soc..

[28] Ubriaco M.R. (2009). Entropies based on fractional calculus. Phys. Lett. A.

[29] Radhakrishnan C., Chinnarasu R., Jambulingam S. (2014). A Fractional Entropy in Fractal Phase Space: Properties and Characterization. Int. J. Stat. Mech..

[30] Karci A. (2016). Fractional order entropy: New perspectives. Optik.

[31] Suthaharan S. (2016). Decision Tree Learning. Machine Learning Models and Algorithms for Big Data Classification.

[32] De la Cruz-García J.S., Bory-Reyes J., Ramirez-Arellano A. (2022). A Two-Parameter Fractional Tsallis Decision Tree. Entropy.

[33] Dempster A.P. (1968). A Generalization of Bayesian Inference. J. R. Stat. Soc. Ser. B Stat. Methodol..

[34] Shafer G. (1976). A Mathematical Theory of Evidence.

[35] Fang L., Yi C., Chong W. An Evidence Theory Decision Tree Algorithm for Uncertain Data. Proceedings of the 2009 Third International Conference on Genetic and Evolutionary Computing.

[36] Peñafiel S., Baloian N., Sanson H., Pino J.A. (2020). Applying Dempster–Shafer theory for developing a flexible, accurate and interpretable classifier. Expert Syst. Appl..

[37] Shannon C.E. (1948). A mathematical theory of communication. Bell Syst. Tech. J..

[38] Weaver W. (1963). The Mathematical Theory of Communication.

[39] Maszczyk T., Duch W. (2008). Comparison of Shannon, Rényi and Tsallis Entropy Used in Decision Trees. Artificial Intelligence and Soft Computing—ICAISC 2008, Proceedings of the 9th International Conference, Zakopane, Poland, 22–26 June 2008.

[40] Downarowicz T. (2011). Entropy in Dynamical Systems.

[41] Rényi A. (1961). On measures of entropy and information. Proceedings of the Fourth Berkeley Symposium on Mathematical Statistics and Probability, Volume 1: Contributions to the Theory of Statistics.

[42] Tsallis C. (1988). Possible generalization of Boltzmann-Gibbs statistics. J. Stat. Phys..

[43] Hasell J. (2023). Measuring Inequality: What Is the Gini Coefficient? Our World in Data. https://ourworldindata.org/what-is-the-gini-coefficient.

[44] Kass G.V. (1980). An Exploratory Technique for Investigating Large Quantities of Categorical Data. Appl. Stat..

[45] R Core Team (2024). R: A Language and Environment for Statistical Computing.

[46] Gajowniczek K., Orłowski A., Ząbkowski T. (2018). Simulation Study on the Application of the Generalized Entropy Concept in Artificial Neural Networks. Entropy.

[47] Gajowniczek K., Karpio K., Łukasiewicz P., Orłowski A., Ząbkowski T. (2015). Q-Entropy Approach to Selecting High Income Households. Acta Phys. Pol. A.

[48] Langer M., Oster D., Speith T., Hermanns H., Kästner L., Schmidt E., Sesing A., Baum K. (2021). What do we want from Explainable Artificial Intelligence (XAI)?—A stakeholder perspective on XAI and a conceptual model guiding interdisciplinary XAI research. Artif. Intell..

[49] Saranya A., Subhashini R. (2023). A systematic review of Explainable Artificial Intelligence models and applications: Recent developments and future trends. Decis. Anal. J..

[50] Machado J.A.T., Galhano A.M.S.F., Trujillo J.J. (2013). On development of fractional calculus during the last fifty years. Scientometrics.

[51] Urbani M., Gasparini G., Brunelli M. (2023). A numerical comparative study of uncertainty measures in the Dempster–Shafer evidence theory. Inf. Sci..

[52] Balakrishnan N., Buono F., Longobardi M. (2022). A unified formulation of entropy and its application. Phys. A Stat. Mech. Its Appl..

[53] Mageed I.A., Zhang Q. An Introductory Survey of Entropy Applications to Information Theory, Queuing Theory, Engineering, Computer Science, and Statistical Mechanics. Proceedings of the 2022 27th International Conference on Automation and Computing (ICAC).

